# Mechanistic framework predicts drug-class specific utility of antiretrovirals for HIV prophylaxis

**DOI:** 10.1371/journal.pcbi.1006740

**Published:** 2019-01-30

**Authors:** Sulav Duwal, Laura Dickinson, Saye Khoo, Max von Kleist

**Affiliations:** 1 Department of Mathematics & Computer Science, Freie Universität Berlin, Germany; 2 Institute of Translational Medicine, University of Liverpool, United Kingdom; ETH Zürich, SWITZERLAND

## Abstract

Currently, there is no effective vaccine to halt HIV transmission. However, pre-exposure prophylaxis (PrEP) with the drug combination Truvada can substantially decrease HIV transmission in individuals at risk. Despite its benefits, Truvada-based PrEP is expensive and needs to be taken once-daily, which often leads to inadequate adherence and incomplete protection. These deficits may be overcome by next-generation PrEP regimen, including currently investigated long-acting formulations, or patent-expired drugs. However, poor translatability of animal- and *ex vivo*/*in vitro* experiments, and the necessity to conduct long-term (several years) human trials involving considerable sample sizes (N>1000 individuals) are major obstacles to rationalize drug-candidate selection. We developed a prophylaxis modelling tool that mechanistically considers the mode-of-action of all available drugs. We used the tool to screen antivirals for their prophylactic utility and identify lower bound effective concentrations that can guide dose selection in PrEP trials. While *in vitro* measurable drug potency usually guides PrEP trial design, we found that it may over-predict PrEP potency for all drug classes except reverse transcriptase inhibitors. While most drugs displayed graded concentration-prophylaxis profiles, protease inhibitors tended to switch between none- and complete protection. While several treatment-approved drugs could be ruled out as PrEP candidates based on lack-of-prophylactic efficacy, darunavir, efavirenz, nevirapine, etravirine and rilpivirine could more potently prevent infection than existing PrEP regimen (Truvada). Notably, some drugs from this candidate set are patent-expired and currently neglected for PrEP repurposing. A next step is to further trim this candidate set by ruling out compounds with ominous safety profiles, to assess different administration schemes in silico and to test the remaining candidates in human trials.

## Author summary

Pre-exposure prophylaxis (PrEP) is a novel, promising strategy to halt HIV transmission. PrEP with Truvada can substantially decrease the risk of infection. However, individuals often inadequately adhere to the once-daily regimen and the drug is expensive. These shortcomings may be overcome by next-generation PrEP compounds, including long-acting formulations. However, poor translatability of animal- and *ex vivo*/*in vitro* experiments, and difficulties in conducting long-term trials involving considerable sample sizes (N > 1000 individuals) make drug-candidate selection and optimization of administration schemes costly and often infeasible. We developed a simulation tool that mechanistically considers the mode-of-action of all antivirals. We used the tool to screen all available antivirals for their prophylactic utility and identified lower bound effective concentrations for designing PrEP dosing regimen in clinical trials. We found that *in vitro* measured drug potency may over-predict PrEP potency, for all antiviral classes except reverse transcriptase inhibitors. We could rule out a number of antivirals for PrEP repurposing and predicted that darunavir, efavirenz, nevirapine, etravirine and rilpivirine provide complete protection at clinically relevant concentrations. Further trimming of this candidate set by compound-safety and by assessing different implementation schemes is envisaged.

## Introduction

Pre-exposure prophylaxis (PrEP) to prevent HIV infection (using drugs which are licensed for its treatment) has been assessed in people at high risk of sexual transmission. Of the available agents, once-daily tenofovir and emtricitabine (Truvada) have been extensively studied, and demonstrate protective efficacy (59–100% [1, 2]) in individuals who are adherent to the medication; conversely poor medication adherence explains the lack of protection observed in some trials [3]. However, major shortcomings of Truvada-based PrEP are its costs [4], a residual infection risk and the necessity for daily drug intake (which often leads to inadequate adherence). These deficits may be overcome by next-generation PrEP regimen, including patent-expired antivirals and long-acting formulations.

Studies assessing next-generation PrEP regimen are underway [5], but rational selection of which agents to advance into PrEP trials based on their intrinsic pharmacology and mode of action has not been comprehensively or systematically undertaken. Moreover, studies have focussed on patent-protected compounds [6], which are likely unaffordable in resource-constrained settings [4] hit hardest by the epidemic.

The considerable sample sizes (*N* > 1000 individuals) and clinical trial duration required (years) to test any new candidate against tenofovir-emtricitabine, and the need to assess regimens with forgiveness for missed dosing or episodic, event-driven PrEP make the current strategy of empirical drug selection costly and prone to failure. We chose to explore an alternative strategy by developing a mathematical modelling tool to assess the per-contact efficacy of anti-HIV drugs. This approach allows prediction of prophylactic utility by integrating drug specific factors (pharmacokinetic/pharmacodynamic (PK/PD) attributes) and attributes of the targeted risk group in order to probe and discard candidates, accelerate drug development and markedly reduce costs. In this work, we are particularly interested in agents where existing patents had already, or are about to expire, in order to maximise the potential impact for low and middle income countries.

Various epidemiological modelling approaches have been used to predict the public health benefits of PrEP [7] and the risk of emergent drug resistance [8–10]. These approaches are highly dependent on *ad hoc* parameter assumptions [11] (specifically the per-contact PrEP efficacy), which may explain the different and contradictory predictions which have emerged.

Knowledge of the per-contact PrEP efficacy, ideally concentration-prophylaxis relationships, are currently lacking and parameters derived from animal models poorly translate into human efficacy. Concentration-prophylaxis relationships are particularly critical to define lower concentrations in human trials that can attain e.g. > 90% protection: I.e., ideally a PrEP candidate should be dosed such that the concentrations stay above this target (e.g. 90% protection) and at the same time avoid adverse effects in all individuals. For prophylaxis, there is a general void of information regarding drug-specific and drug-class specific concentration-prophylaxis relationships. While the potency of drugs to inhibit HIV replication can readily be measured *in vitro*, researchers are often unaware that this measure of drug potency may not coincide with the potency to prevent HIV infection (prophylactic potency) and consequently PrEP trial design may be flawed, incurring costs and putting individuals at risk.

In a top-down approach, Hendrix et al. [12] analyzed available clinical data for Truvada to define concentration-prophylaxis relationships. However, this approach is naturally limited to PrEP candidates where sufficient clinical data already exists and is not able to disentangle the potency of the administered drugs from confounding factors. More mechanistic, bottom-up approaches integrate various host- and viral factors [13–18] to predict the probability of viral extinction. Despite their advantages, these approaches conventionally do not establish concentration-prophylaxis relations, or they are specific to particular drugs [17] or drug classes [18].

In this work, we will first analyze the drug-class specific relation between *in vitro* potency and PrEP efficacy and its dependency on the amount- and type of transmitted virus. Utilizing pharmacokinetic and pharmacodynamic data for all treatment-approved drugs, and simulating typical viral exposures during sexual contact, we will then screen all treatment-approved drugs for their PrEP utility and assess the sensitivity of the prophylactic endpoint with regard to uncertainties in viral dynamics parameters and with regard to variabilities in drug concentration, which can typically result from inter-individual metabolic differences or differences in medication adherence. Our central aim is to provide a tool to screen out drug candidates with a lack of- or uncertain prophylactic efficacy.

## Methods

Before HIV infection is irreversibly established, viral replication is highly stochastic [19], corroborated by the observation of a low transmission probability per exposure [20, 21] and a low number of founder viruses responsible for establishing infection [22–25]. The stochasticity can be explained by the order in which viral dynamics reactions occur: For example, when a single virus comes into proximity of target cells, it may either be cleared or it may infect the target cell which can eventually lead to systemic infection. In the current work, we will make use of branching process theory [26] to derive analytical solutions for the probability of viral extinction [13, 15], i.e. the probability to hit the absorbing state where all viral compartments go extinct. These solutions can be used directly to benchmark antivirals for their potential to prevent infection as exemplified in the current work, or they can be used to design efficient algorithms for the numerically *exact* simulation of complex prophylactic dosing regimen as proposed in a related article [27].

### Prophylactic efficacy

The infection probability *P*_I_(*Y*_0_) for some initial state *Y*_0_ is the complement of the extinction probability *P*_E_(*Y*_0_)

$$\begin{array}{c} P_{I}\left(Y_{0}\right)=1-P_{E}\left(Y_{0}\right), \end{array}$$ (1)

where *Y*_0_ denotes the initial viral population in a replication enabling (target-cell) environment. Throughout the article we will use *Y* = [V, T_1_, T_2_]^*T*^, i.e. the state of the viral dynamics is defined by infectious viruses, early- and productively infected cells as outlined below. The extinction probability is defined by

$$\begin{array}{c} P_{E}\left(Y_{0}\right)≔P(Y_{t}=[\begin{array}{c} 0\\ 0\\ 0 \end{array}]\;|\;Y_{0}=[\begin{array}{c} V\\ T_{1}\\ T_{2} \end{array}]) \end{array}$$ (2)

for *t* → ∞. In words, the probability that all viral compartments will eventually go extinct. The prophylactic efficacy *φ* then denotes the reduction in infection probability *per contact*,

$$\begin{array}{c} \varphi =1-\frac{P_{I}\left(Y_{0}|D\right)}{P_{I}\left(Y_{0}|⌀\right)}\;\;\left(\text{prophylactic}\;\text{efficacy}\right), \end{array}$$ (3)

where *P*_I_(*Y*_0_|*D*) and *P*_I_(*Y*_0_|⌀) denote the infection probabilities in the presence- and absence of prophylactic drugs *D* respectively. The term *P*_I_(*Y*_0_|*D*) was computed using a mathematical model of the viral dynamics (below) and by mechanistically considering the *direct* effects of the distinct antivirals on viral replication whereas *P*_I_(*Y*_0_|⌀) is computed analogously, assuming the absence of drug *D* = 0.

### Drug-class specific *direct* effects on virus replication

#### Virus replication dynamics

**Fig 1:**
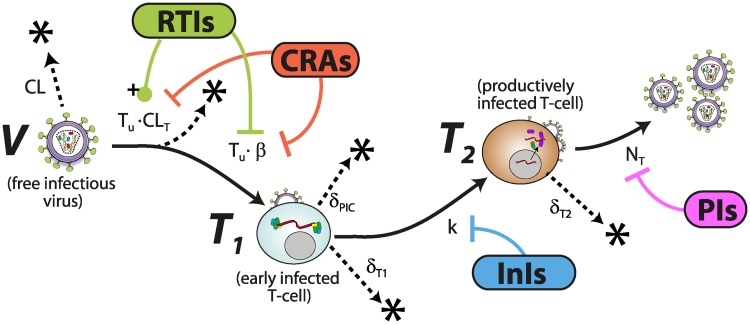
Schematic of the HIV replication cycle and mechanism of interference by treatment-approved drug classes. Free viruses are cleared by the immune system with a rate constant CL. Further, free viruses can be also cleared during unsuccessful T-cell infection CL_T_ through the destruction of essential viral components of the reverse transcription-, or pre-integration complex [37, 38]. The term *β* represents the lumped rate of infection of T-cells, including the processes of virus attachment to the cell, fusion and reverse transcription, leading to an early infected cell T_1_, before proviral integration. Similarly, the term *k* denotes the rate by which early infected T_1_ cells are transformed into productively infected T_2_ cells, involving proviral integration and cellular reprogramming. The term N_T_ denotes the rate of production of infectious virus progeny by productively infected T_2_ cells. The rates *β*, CL_T_, *k* and N_T_ may be modified by different antiretrovirals as indicated by bars (inhibition) and pointers with plus sign (drug-dependent increase). The terms $\delta_{T_{1}}<\delta_{T_{2}}$ denote the rates of clearance of T_1_ and T_2_ cells respectively and *δ*_PIC_ denotes the rate of intracellular destruction of the pre-integration complex. CRA: Co-receptor antagonists, RTIs: reverse transcriptase inhibitors, InIs: Integrase inhibitors, PIs: Protease inhibitors.

We adopted the viral dynamics model described in [28, 29]. Although this model is a coarse representation of the molecular events happening during virus replication, it allows to accurately and mechanistically describe the effect of all existing antiretroviral drug classes on viral replication, as demonstrated in e.g. [30], and can be parameterized by available *in vitro* and *clinical* data. Unlike the original model [28, 29] we do not consider macrophages, motivated by the observation that transmitted viruses are not macrophage-tropic [31, 32] and in line with related modelling approaches [13, 14, 33–35]. The model is schematically depicted in Fig 1. The modelled viral replication cycle consists of free infectious viruses, uninfected T-cells, early infected T-cells (T_1_) and productively infected T-cells (T_2_). Early infected T-cells (T_1_) and productively infected T-cells (T_2_) denote T-cells prior- and after proviral integration respectively, where the latter produces virus progeny. The term T_u_ = λ_T_/*δ*_T_ denotes the steady state level of uninfected T-cells prior to virus challenge, where λ_T_ denotes the birth and *δ*_T_ the death rate of uninfected T-cells. During the onset of infection the number viruses are relatively low and the number of uninfected T-cells is fairly unaffected by virus dynamics [33, 36]. Thus, for all computations, we consider the number of uninfected T-cells to be constant, in line with related approaches [14, 15]. The dynamics of the stochastic viral replication model after virus exposure are then defined by six reactions. In absence of antivirals ⌀ we have

$$\begin{array}{ccc} a_{1}\left(⌀\right)= & (\text{CL}+{\text{CL}}_{T}\cdot T_{u})\cdot V & \left(\text{clearance}\;\text{of}\;\text{free}\;\text{virus;}\;V\to *\right) \end{array}$$ (4)

$$\begin{array}{ccc} a_{2}\left(⌀\right)= & (\delta_{\text{PIC}}+\delta_{T_{1}})\cdot T_{1} & \left(\text{clearance}\;\text{of}\;\text{early}\;\text{infected}\;\text{cell;}\;T_{1}\to *\right) \end{array}$$ (5)

$$\begin{array}{ccc} a_{3}\left(⌀\right)= & \delta_{T_{2}}\cdot T_{2} & \left(\text{clearance}\;\text{of}\;\text{late}\;\text{infected}\;\text{cell;}\;T_{2}\to *\right) \end{array}$$ (6)

$$\begin{array}{ccc} a_{4}\left(⌀\right)= & \beta \cdot T_{u}\cdot V & \left(\text{successful}\;\text{infection}\;\text{of}\;\text{a}\;\text{suscept.}\;\text{cell;}\;V\to T_{1}\right) \end{array}$$ (7)

$$\begin{array}{ccc} a_{5}\left(⌀\right)= & k\cdot T_{1} & \left(\text{proviral}\;\text{integration;}\;T_{1}\to T_{2}\right) \end{array}$$ (8)

$$\begin{array}{ccc} a_{6}\left(⌀\right)= & N_{T}\cdot T_{2} & (\text{production}\;\text{of}\;\text{infectious}\;\text{virus;}\;T_{2}\to V+T_{2}), \end{array}$$ (9)

with ${CL}_{T}=\left(\frac{1}{\rho_{\text{rev},⌀}}-1\right)\cdot \beta$ in Eq (4) as outlined in [28] where *ρ*_*rev*,⌀_ = 0.5 denotes the probability to successfully complete reverse transcription in the absence of inhibitors [37, 38]. Free viruses are cleared by the immune system with a rate constant CL. Further, free viruses can be also cleared during unsuccessful T-cell infection CL_T_ through the destruction of essential viral components of the reverse transcription-, or pre-integration complex intracellularly after the virus entered the cell [37, 38]. The term *β* represents the lumped rate of infection of T-cells, including the processes of virus attachment to the cell, fusion and reverse transcription, leading to an early infected cell T_1_, before proviral integration. Similarly, the term *k* denotes the rate by which early infected T_1_ cells are transformed into productively infected T_2_ cells, involving proviral integration and cellular reprogramming. The term N_T_ denotes the rate of production of infectious virus progeny by productively infected T_2_ cells (*infectious burst size)*. The terms $\delta_{T_{1}}<\delta_{T_{2}}$ denote the rates of clearance of T_1_ and T_2_ cells respectively and *δ*_PIC_ denotes the rate of intracellular destruction of the pre-integration complex. Parameters for the viral model are summarized in Table 1 and a mechanistic derivation of the dynamics from first principles is given in [28] (Supplementary Text therein).

**Table 1:** Parameters generally used for the viral dynamics model. Excerpt from [28], except for CL(naive), which assumed that virus clearance is smaller in virus-naive individuals compared to infected individuals, in line with [17, 72]. All parameters refer to the absence of drug treatment ⌀. All parameters in units [1/day], except for λ [cells/day] and *β* [1/day/virus]. Parameter sensitivity was assessed in S2 Text.

Parameter	Value	Reference	Parameter	Value	Reference
λ_T_	2⋅10^9^	[78]	*k*	0.35	[38]
$\delta_{T},\delta_{T_{1}}$	0.02	[79]	*β*	8⋅10^−12^	[80]
$\delta_{T_{2}}$	1	[81]	N_T_	670	[28, 79]
*δ*_PIC_	0.35	[38, 82]	CL(naive)	2.3	[14, 33]

#### Class-specific *direct* drug effects

The *direct* effect of drugs *D* ∈ {RTI, CRA, InI, PI} on their target process is typically modelled using the Emax-equation [39]

$$\begin{array}{c} \eta_{D}\left(t\right)=\frac{D_{t}^{m}}{{\text{IC}}_{50}^{m}+D_{t}^{m}}, \end{array}$$ (10)

where *D*_*t*_ is the target site concentration of the drug and the term IC_50_ and *m* denote the drug concentration at which the targeted process is inhibited by 50% and a hill coefficient [40] respectively. In the current article we will assume that drug concentrations stay constant over the course of infection, which allows to study drug- and drug class specific properties with regard to prophylaxis. This assumption is overcome in related article [27], where pharmacokinetic inputs are explicitly considered to evaluate particular prophylactic dosing regimen.

#### Reverse transcriptase inhibitors

In the presence of reverse transcriptase inhibitors RTI the reaction propensities *a*_1_ and *a*_4_ are affected [28], i.e.

$$a_{1}\left(\text{RTI}\right)=(\text{CL}+(\frac{1}{\rho_{rev,⌀}}-\left(1-\eta_{\text{RTI}}\right))\cdot \beta \cdot T_{u})\cdot V$$ (11)

$$a_{4}\left(\text{RTI}\right)=\left(1-\eta_{\text{RTI}}\right)\cdot \beta \cdot T_{u}\cdot V$$ (12)

where *η*_RTI_ ∈ [0, 1] follows from Eq (10). Eq (11) results from the specific action of reverse transcriptase inhibitors: they act only after irreversible fusion of viral particles and release of viral contents has occurred by halting reverse transcription, which increases the probability that essential viral constituents get cleared intracellularly preventing viral replication to progress. Thus, inhibition by RTIs can lead to an increase of cell-dependent clearance of viral particles as modelled in Eq (11) (see Supplementary Information of [28] for an explicit derivation). From the equations it becomes evident that the increase in cell-dependent clearance (effect on *a*_1_) matches the reduction in successful infection (effect on *a*_4_). The validity of this model has been assessed in [30].

#### Other inhibitor classes

Co-receptor antagonists (CRA) decrease the infection propensity *a*_4_ and *a*_1_, whereas integrase inhibitors InI decrease *a*_5_ and protease inhibitors PI reduce *a*_6_ respectively by a factor (1 − *η*_*D*_) [28]:

$$a_{1}\left(\text{CRA}\right)=(\text{CL}+\left(1-\eta_{\text{CRA}}\right)\cdot (\frac{1}{\rho_{\text{rev},⌀}}-1)\cdot \beta \cdot T_{u})\cdot V$$ (13)

$$a_{4}\left(\text{CRA}\right)=\left(1-\eta_{\text{CRA}}\right)\cdot \beta \cdot T_{u}\cdot V$$ (14)

$$a_{5}\left(\text{InI}\right)=\left(1-\eta_{\text{InI}}\right)\cdot k\cdot T_{1}$$ (15)

$$a_{6}\left(\text{PI}\right)=\left(1-\eta_{\text{PI}}\right)\cdot N_{T}\cdot T_{2}$$ (16)

Note that unlike RTIs, CRAs decrease the adsorption of viral particles by cells, which does not *per se* lead to a cell-dependent clearance of viral particles as in the case of RTIs. InIs block proviral integration, affecting *a*_5_ and PIs prevent maturation, which lowers the amount of *infectious* viruses produced.

### Probability of virus extinction

For the ease of notation we introduce the unit vectors $\hat{V}$, ${\hat{T}}_{1}$ and ${\hat{T}}_{2}$ which represent the states where only one infected compartment is present (either virus, early- or late infected cells)

$$\begin{array}{c} \hat{V}=[\begin{array}{c} 1\\ 0\\ 0 \end{array}]\;,\;{\hat{T}}_{1}=[\begin{array}{c} 0\\ 1\\ 0 \end{array}]\;,\;{\hat{T}}_{2}=[\begin{array}{c} 0\\ 0\\ 1 \end{array}] \end{array}$$ (17)

While free virus is typically transmitted, our framework also allows to study prophylactic efficacy for arbitrary initial states. Using the notation above, any state of the system can be expressed as a linear combination of the unit vectors above. For example, $5\cdot \hat{V}⊕3\cdot {\hat{T}}_{1}⊕12\cdot {\hat{T}}_{2}$ denotes the state where we have 5 viruses, 3 early infected cells and 12 late infected cells. In S1 Text we provide a detailed derivation of infection/extinction probabilities after viral exposure. Herein, we will provide a sketch of the central idea.

Starting from a single virus $Y_{0}=\hat{V}$, we can write the Chapman-Kolmogorov equation:

$$\begin{array}{ccc} P_{E}\left(Y_{0}=\hat{V}\right) & = & \sum_{n=0}^{\infty }P\left(Y_{r}=n\cdot \hat{V}|Y_{0}=\hat{V}\right)\cdot P_{E}{\left(Y_{r}=\hat{V}\right)}^{n}. \end{array}$$ (18)

In words, the extinction probability $P_{E}(Y_{0}=\hat{V})$ is given by the probability that *n* viruses are produced in a single replication cycle *r*, $\mathbb{P}(Y_{r}=n\cdot \hat{V}|Y_{0}=\hat{V})$, and that all of these viruses *eventually* go extinct, considering all possible values of *n*. Herein we assumed *statistical independence*, i.e. $P_{E}(Y_{r}=n\cdot \hat{V})=P_{E}(Y_{r}=\hat{V}{)}^{n}$. Furthermore, the extinction probabilities for parent- and progeny virus are identical when the inhibitor efficacy is constant, i.e. $P_{E}(Y_{0}=\hat{V})=P_{E}(Y_{r}=\hat{V})$. Next, we construct the embedded Markov chain [26] corresponding from the continuous-time Markov jump model depicted in Fig 1 with parameters in Table 1 (details in S1 Text). This allows to derive algebraic formulas for $\mathbb{P}(Y_{r}=n\cdot \hat{V}|Y_{0}=\hat{V}),\;n=0\ldots \infty$. Substituting these into Eq (18), rearranging and solving for $P_{E}(Y_{0}=\hat{V})$ yields a quadratic formula. Solving the quadratic formula, and using *P*_E_(⋅) = 1 − *P*_I_(⋅) we derive analytical solutions for the infection probabilities after exposure to a single virus $\hat{V}$, early- ${\hat{T}}_{1}$ and late infected cell ${\hat{T}}_{2}$:

$$P_{I}\left(Y_{0}=\hat{V}\right)=\text{max}(0,\frac{a_{4}\left(D\right)}{a_{1}\left(D\right)+a_{4}\left(D\right)}\cdot \frac{a_{5}\left(D\right)}{a_{2}+a_{5}\left(D\right)}(1-\frac{1}{R_{0}\left(D\right)}))$$ (19)

$$P_{I}\left(Y_{0}={\hat{T}}_{1}\right)=\text{max}(0,\frac{a_{5}\left(D\right)}{a_{2}+a_{5}\left(D\right)}\cdot (1-\frac{1}{R_{0}\left(D\right)}))$$ (20)

$$P_{I}\left(Y_{0}={\hat{T}}_{2}\right)=\text{max}(0,1-\frac{1}{R_{0}\left(D\right)}).$$ (21)

where *R*_0_(*D*) denotes the basic reproductive number, i.e. the average number of viruses produced from a single founder virus [41] in a single replication cycle under the action of drug *D*. Using our model we have $R_{0}(D)=\frac{a_{4}(D)}{a_{1}(D)+a_{4}(D)}\cdot \frac{a_{5}(D)}{a_{2}+a_{5}(D)}\cdot \frac{a_{6}(D)}{a_{3}}$. The first solution *P*_I_(⋅) = 0 of Eqs (19)–(21) are valid in the regimen where *R*_0_(*D*) ≤ 1, i.e. in the regimen where extinction is certain. The second solution describes the case where infection may occur, i.e. *R*_0_(*D*) > 1. The pre-terms in the second solution of Eqs (19) and (20) denote the bottlenecking probabilities that a late-infected, virus producing cell is reached, starting from a free virus (Eq (19)) or starting from an early infected cell (Eq (20)) respectively.

We can assume *statistical independence* during the onset of infection (i.e. competition for target cells is negligible) as noted before. Hence, for any given combination of free virus, early-stage infected cell and late-stage infected cell the extinction probability is given by

$$\begin{array}{ccc} P_{E}(Y_{0}=[\begin{array}{c} V\\ T_{1}\\ T_{2} \end{array}]) & = & {\left(P_{E}\left(Y_{0}=\hat{V}\right)\right)}^{V}\cdot {\left(P_{E}\left(Y_{0}={\hat{T}}_{1}\right)\right)}^{T_{1}}\cdot {\left(P_{E}\left(Y_{0}={\hat{T}}_{2}\right)\right)}^{T_{2}}, \end{array}$$ (22)

where the exponents V, T_1_ and T_2_ denote the number of free virus, early- and late-stage infected cells present and where we notice that *P*_E_(⋅) = 1 − *P*_I_(⋅).

### Virus exposure model

Initial viral exposure after sexual intercourse occurs at tissue sites typically not receptive for establishing and shedding HIV infection (e.g. mucosal tissues). Hence, the virus needs to pass several bottlenecks and physiological barriers to reach a replication enabling (target-cell) environment where infection can be established and from where it can shed systemically [42]. To determine realistic inoculum sizes after sexual exposure to HIV, we previously developed a data-driven statistical model linking plasma viremia in a transmitter to the initial viral population *Y*_0_ in a replication-enabling environment [18] (Supplementary Note 4 therein for details). Herein, we used the ‘exposure model’ to compute drug efficacy estimates after homosexual exposure presented in section *Prophylactic efficacy of treatment-approved antivirals*. In brief, this ‘exposure model’ was developed to capture key clinical observations: (i) the average HIV transmission probabilities per exposure as reported in [20, 21, 43]. (ii) the fact that viral loads in the untreated transmitter population are approximately log-normal distributed [18, 44–46] (*μ* = 4.51, *σ* = 0.98) and (iii) the observation that the plasma viremia in the transmitter is the most dominant factor determining HIV transmission [44, 47–49]. More specifically, it was reported that each 10-fold increase in the transmitter’s viral load increases the transmission probability per coitus by approximately 2.45-fold [47] (similar values confirmed in [49]). The aforementioned clinical observations can be summarised in the formula below:

$$\begin{array}{c} {\bar{P}}_{\text{trans}}=\int_{\nu =0}^{\infty }P\left(\text{VL}=\nu \right)\cdot (\sum_{n=0}^{\infty }P\left(Y_{0}=n\cdot \hat{V}|\text{VL}=\nu \right)\cdot P_{I}\left(Y_{0}=n\cdot \hat{V}\right)) \end{array}$$ (23)

where ${\bar{P}}_{trans}$ is the average transmission probability per exposure/coitus (given in (i)), *P*(VL = *ν*) is the probability density of viral load in the donor (log-normal distributed, given in (ii)), $P_{I}(Y_{0}=n\cdot \hat{V})$ is the infection probability when *n* viruses reach a replication enabling site (computed from the virus dynamics model above with $P_{I}(Y_{0}=\hat{V})\approx 0.0996$) and $P(Y_{0}=n\cdot \hat{V}|VL=\nu )$ denotes the ‘exposure model’ (the probability that *n* viruses reach a replication-enabling compartment after viral exposure from a transmitter with virus load *ν*). For the ‘exposure model’, we assumed a binomial distribution

$$\begin{array}{c} P\left(Y_{0}=n\cdot \hat{V}|\text{VL}=\nu \right)=(\begin{array}{c} \left\lceil \nu^{m}\right\rceil \\ n \end{array})\cdot r^{n}\cdot {\left(1-r\right)}^{\left\lceil \nu^{m}\rceil -n\right)} \end{array}$$ (24)

where *m* = log_10_ (2.45) is given by (iii) [47] and the *success probability* *r* was estimated in a previous work [18] (Supplementary Note 4 therein), e.g. *r*_homo_ = 3.71 ⋅ 10^−3^ for homosexual exposure. However, the model can be adapted to the different exposure types (e.g. heterosexual, needle-stick, etc …). In this model, the *success probability r* summarises both the extent of local exposure, as well as the probability of passing all bottlenecking physiological barriers and reaching a replication enabling target cell compartment. Lastly, in line with Keele et al. [22], we observed that if infection occurs in our model it is established by a very low number of viruses after homosexual contact and usually by a single founder virus after heterosexual contact.

## Results

### Relation between direct effects and prophylactic efficacy

Drug-specific inhibition of viral replication can be studied *in vitro*, for example in single-round turnover experiments [40] or even more mechanistically using enzymatic assays in conjunction with appropriate mathematical models [50]. Since the infection risk per exposure is already low in untreated individuals [20, 21], exploring the prophylactic efficacy (reduction in infection risk) in the clinic is difficult, requiring very long (several years) clinical trials with many individuals (*N* > 1000) to achieve statistically evaluable results. Systematic evaluation of concentration-effect relations is not feasible in this context, notwithstanding ethical concerns.

**Fig 2:**
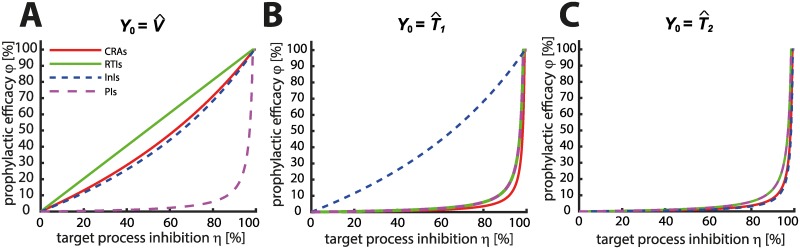
Relation between direct drug effect and prophylactic efficacy. The relation between direct drug effect *η* and prophylactic efficacy *φ* (reduction in infection) is shown for different drug classes utilizing the viral model depicted in Fig 1 with parameters stated in Table 1. Panel **A**: Relation between *η* and *φ* when a single virus $Y_{0}=\hat{V}$ reached a replication-enabling compartment in the virus-exposed individual. Panel **B**: Relation between *η* and *φ* when a single early infected cell $Y_{0}={\hat{T}}_{1}$ or (panel **C**) a late infected T-cell $Y_{0}={\hat{T}}_{2}$ reached a replication-enabling compartment. Solid red lines: CRAs, solid green line: RTIs, dashed blue line: InI, dashed purple line: PIs.

We wanted to gain a deeper insight how *in vitro* measurable direct drug efficacy *η* translates into prophylactic efficacy *φ* (reduction in infection probability per exposure) in a drug-class specific manner. Particularly, since different antiviral drug classes inhibit distinct stages in the HIV replication cycle, we wanted to elucidate how these different mechanisms of action affect prophylaxis. We combined Eqs (11)–(16) with Eqs (19)–(21) into Eq (3) to predict prophylactic efficacy. When relating *direct* drug effects *η* to prophylactic efficacy *φ* we observed striking drug-class specific differences as illustrated in Fig 2. Using parameters from Table 1 we found that the prophylactic efficacy *φ* may be less than predicted by *in vitro* measurable direct drug effects *η*. The sole exception are reverse transcriptase inhibitors (RTI) in case of exposure to a single virus particle $Y_{0}=\hat{V}$ where the two measures of drug efficacy coincide. While the prophylactic efficacy after exposure to a single virus are moderately less than the direct effects of co-receptor antagonists CRA and integrase inhibitors InI respectively (Fig 2A), there is a profound difference for protease inhibitors, which do not seem to reduce HIV transmission unless their direct efficacy *η* exceeds ≈ 95%. Interestingly, a similar observation using a different mathematical model and only distinguishing RTIs and PIs has been made by Conway et al. [13].

While HIV-transmission typically occurs after exposure to free virus, it is still useful to study the prophylactic efficacy of distinct drug classes in the hypothetical case when infected cells were present in the exposed individual. A realistic example for this scenario is post-exposure prophylaxis (PEP): During PEP, drugs are taken shortly *after* virus exposure and initial viral replication steps may have taken place generating early- or late infected cells. As can be seen in Fig 2B and 2C, the prophylactic efficacy of all drugs profoundly deteriorates compared to their direct effects, i.e. only very effective (in terms of *η*) drugs may prevent systemic infection once cells become infected in the exposed individual. An exception are integrase inhibitors: their prophylactic efficacy *φ* is moderately less than their direct effect *η* (panel B) if only early infected cells T_1_ (before proviral integration) were present. Thus, while the prophylactic efficacy of all other drug classes is profoundly less than their direct effects once infected cells emerged, integrase inhibitors may still potently prevent infection. An intuitive explanation for the deterioriation of prophylactic efficacy can be made in terms of changes in drug-target stoichiometry: For example, after exposure to a single virus $\hat{V}$, drugs from the classes of CRAs, RTIs and InIs need to block a single reaction to foster viral extinction. For PIs however, the same is only achieved if maturation of the entire viral progeny is inhibited (possibly hundreds of particles). Similarly, when considering a single early infected cell ${\hat{T}}_{1}$, CRAs and RTIs can only prevent further viral expansion *after* viral progeny has emerged. Subsequently, for each viral particle (possibly hundreds) the respective target processes (receptor binding, reverse transcription) need to be blocked by the inhibitors. Along the same lines of argumentation it is also evident that prophylactic efficacy is generally more favourable in the case of PrEP, compared to post-exposure prophylaxis (PEP), where initial viral replication may have occurred.

### *In vitro* drug potency may overestimate PrEP potency

*In vitro* measured drug potency IC_50_, IC_90_ usually guides the design of PrEP trials [51]. In particular, dosing regimen are designed so that the majority of individuals achieve drug levels just above the 90% inhibitory concentrations IC_90_. However, it has never been rigorously investigated whether these ‘target concentrations’ are sufficient to provide 90% protection against HIV infection. Integrating Eq (10) into Eqs (11)–(16), (19) and (3) allows to predict the concentration-prophylaxis profile for different HIV-1 inhibitor classes. Rearranging this composite equation reveals how *in vitro* measured drug potency IC_50_, IC_90_ can be translated into prophylactic potency (50% and 90% reduction in infection risk, EC_50_ and EC_90_, respectively), guiding clinical trial design. The derived analytical expressions for the prophylactic efficacy (reduction in infection risk) indicate that the shape of the concentration-prophylaxis profile varies considerably for different HIV-1 inhibitor classes with important consequences for their prophylactic endpoints (% reduction in HIV transmissibility).

After exposure to a single virion $Y_{0}=\hat{V}$, the overall shape of the concentration-prophylaxis profile for co-receptor antagonists (CRAs), reverse transcriptase inhibitors (RTIs) and integrase inhibitors (InIs) is a classical Emax equation (the equation of choice for evaluating concentration-effect relations), see S1 Text for derivation.

$$\varphi (\hat{V})=\frac{R_{0}\left(⌀\right)}{R_{0}\left(⌀\right)-1}\cdot \frac{D^{m}}{{\text{IC}}_{50}^{m}(\frac{1}{\upsilon })+D^{m}}\;\overset{R_{0}\left(⌀\right)\gg 1}{\approx }\frac{D^{m}}{{\text{EC}}_{50}^{m}+D^{m}}\;\;\text{(CRA)}$$ (25)

$$\varphi (\hat{V})=\frac{R_{0}\left(⌀\right)}{R_{0}\left(⌀\right)-1}\cdot \frac{D^{m}}{{\text{IC}}_{50}^{m}+D^{m}}\;\overset{R_{0}\left(⌀\right)\gg 1}{\approx }\frac{D^{m}}{{\text{EC}}_{50}^{m}+D^{m}}\;\;\text{(RTI)}$$ (26)

$$\varphi (\hat{V})=\frac{R_{0}\left(⌀\right)}{R_{0}\left(⌀\right)-1}\cdot \frac{D^{m}}{{\text{IC}}_{50}^{m}(\frac{1}{\vartheta })+D^{m}}\;\overset{R_{0}\left(⌀\right)\gg 1}{\approx }\frac{D^{m}}{{\text{EC}}_{50}^{m}+D^{m}}\;\;\;\text{(InI)}$$ (27)

where *D* denotes the concentration of the drug in the blood plasma, *m* is a slope parameter and IC_50_ denotes the plasma concentration of the drug that inhibits the targeted process (co-receptor binding, reverse transcription or proviral integration) by 50 percent. This parameter can typically be measured *in vitro*, e.g. using single-round turnover experiments [40] and is stated in Table 2 for various drugs. Parameters $\upsilon =\frac{CL\cdot \rho_{\text{rev},⌀}}{CL\cdot \rho_{\text{rev},⌀}+\beta \cdot T_{u}}<1$ and $\vartheta =\frac{\delta_{PIC}+\delta_{T_{1}}}{\delta_{PIC}+\delta_{T_{1}}+k}<1$ denote the respective probabilities, in the absence of drugs, that the virus is eliminated before entering a host cell, and that essential virus compartments get cleared intracellularly after reverse transcription and before provirus integration. The parameter EC_50_ denotes the plasma concentration of the drug that decreases the probability of infection by 50%, i.e. the *prophylactic potency* of the drug. *R*_0_(⌀) denotes the basic reproductive number in the absence of drugs, i.e. the average number of viruses produced from a single founder virus [41] in a single replication cycle when no antivirals were present (*R*_0_(⌀) ≈ 67 according to the utilized model). When the target cell density is sufficiently high (herein considered as a target cell environment), we have *R*_0_(⌀) ≫ 1 and hence the left-side scaling factor in Eqs (25)–(27) will be close to one, *R*_0_(⌀)/(*R*_0_(⌀) − 1) ≈ 1. An analysis with low target cell densities is provided in S2 Text.

**Table 2:** Pharmacodynamic and pharmacokinetic parameters. IC_50_ [nM] and *m* [unit less] values are available from single turnover experiments in primary peripheral blood mononuclear cells supplemented with 50% human serum from Shen et al. [40], Laskey et al. [92] (DTG) and Jilek at al. [93] (MVC). Because some compounds are highly protein bound, IC_50_ values had to be adjusted for protein binding as outlined in the S5 Text. Indicated values are after protein adjustment. IC_50_ values are reported to be log normal distributed and *m* values to be normal distributed [40, 93] with respective coefficients of variation *CV* = 100 ⋅ *σ*/*μ* [%]. Parameters *C*_min_ and *C*_max_ refer to the minimum and maximum concentrations in [nM] during chronic administration using the standard dosing regimen, taken from Shen et al. [40] except those for DTG [94], RPV [95] and MVC [96] (150mg twice daily). *t*_1/2_—half life of the drug in [hr], *f*_*b*_—fraction of the drug bound to plasma proteins in [%]. ^+^These values were fixed to the typical parameter distributions observed for all other compounds. ^⋄^Parameters were taken from Drug Bank when available https://www.drugbank.ca/, accession numbers: DB04835, DB00625, DB00238, DB00705, DB08864, DB06817, DB09101, DB08930, DB01072, DB00701, DB01264, DB00224, DB00220, DB00932 or ^♭^PubChem https://pubchem.ncbi.nlm.nih.gov, id: 92727. When parameters were not readily available in these databases, parameters were obtained from the indicated literature source. MVC -maraviroc, EFV -efavirenz, NVP -nevirapine, DLV -delavirine, ETR -etravirine, RPV -rilpivirine, RAL -raltegravir, EVG -elvitegravir, DTG -dolutegravir, ATV -atazanavir, APV -amprenavir, DRV -darunavir, IDV -indinavir, LPV -lopinavir, NFV -nelfinavir, SQV -saquinavir, TPV -tipranavir.

Class	Name	IC_50_	(*CV*)	m	(*CV*)	*C*_min_	*C*_max_	*f*_*b*_	*t*_1/2_
CRA	MVC	5.06	(290)	0.61	(27.9)	45	557	76^⋄^	14^⋄^
RTI	EFV	10.7	(16.7)	1.69	(4.73)	5630	12968	99.4 [83]	40^⋄^
RTI	NVP	116	(31.2)	1.55	(9.68)	10883	25153	60^⋄^	45^⋄^
RTI	DLV	336	(44.7)	1.56	(11.5)	10672	27134	98 [84]	5.8^⋄^
RTI	ETR	8.59	(16.3)	1.81	(12.7)	688	1617	99.9 [85]	35 [86]
RTI	RPV	7.73	(17.9)	1.92	(10.4)	177	470	99.1^⋄^	44.5^⋄^
InI	RAL	25.5	(12.1)	1.1	(4.55)	203	3996	83^⋄^	9^⋄^
InI	EVG	55.6	(43.8)	0.95	(4.21)	301	1661	99^⋄^	8.7^⋄^
InI	DTG	89.0	(25.3^+^)	1.3	(15.4^+^)	2918	8471	98.9^⋄^	14.5 [87]
PI	ATV	23.9	(11.8)	2.69	(10.4)	899	6264	86 [88]	7^⋄^
PI	APV	262	(12.6)	2.09	(6.70)	2870	14319	90^⋄^	7.1^⋄^
PI	DRV	45.0	(21.6)	3.61	(8.86)	5081	14783	95 [85]	15^⋄^
PI	IDV	130	(11.0)	4.53	(7.94)	1827	12508	60 [89]	1.8^⋄^
PI	LPV	70.9	(20.1)	2.05	(5.85)	8757	15602	99 [60]	2.5^♭^
PI	NFV	327	(26.8)	1.81	(12.7)	2285	5104	98^⋄^	3.5^⋄^
PI	SQV	88.0	(9.7)	3.68	(6.25)	897	13282	97 [90]	3.9 [91]
PI	TPV	483	(18.0)	2.51	(14.3)	35598	77585	99.9^⋄^	5^⋄^

**Fig 3:**
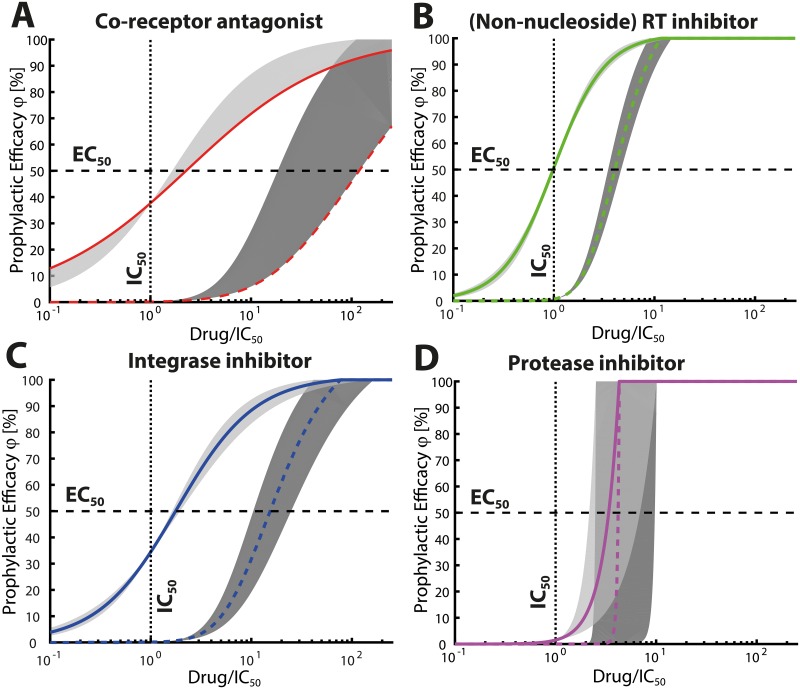
Shape of the concentration-prophylaxis profile. Colored lines depict the concentration-prophylaxis profile for an average drug class-specific slope parameter $\bar{m}$ in Eq (10). Solid colored line for an inoculum of one virus $Y_{0}=\hat{V}$ and dashed colored line for an inoculum of $Y_{0}=100\cdot \hat{V}$. Shaded areas indicate the concentration-prophylaxis profile for the smallest *m*_min_ and largest class-specific slope parameter *m*_max_ for the respective drug class as indicated in Table 2. **A**: Co-receptor antagonists. Currently only one co-receptor antagonist, maraviroc, is approved. We use $\bar{m}=m_{min}=0.61$ and also plot *m*_max_ = 1 as a reference. **B**: Non-nucleoside reverse transcriptase inhibitors (NNRTIs); $\bar{m}=1.71$, *m*_min_ = 1.55 and *m*_max_ = 1.92. Nucleoside reverse transcriptase inhibitors (NRTI) have been analyzed in [18]. **C**: Integrase inhibitors, $\bar{m}=1.12$, *m*_min_ = 0.95 and *m*_max_ = 1.3. **D**: Protease inhibitors; $\bar{m}=2.87$, *m*_min_ = 1.81 and *m*_max_ = 4.53. Utilized virus dynamics parameters are stated in Table 1.

In case of exposure to a single virus particle $\hat{V}$, the slope parameters in the right-most equations coincide with the slope parameter for the respective drug-targeted process *m* (Eq (10)), stated in Table 2. Notably, for RTIs, we have EC_50_ ≈ IC_50_, i.e. the drugs potency measured *in vitro* in single-round turnover experiments [40] directly translates into its potency to prevent infection. Using parameters from Table 1 we observe EC_50_ > IC_50_ for CRAs and InIs, i.e. compared to their *in vitro* potency, they are less potent in preventing infection. This is largely due to the respective factors *ϑ*^−1^, *υ*^−1^ > 1, compare Fig 3A–3C. For InIs this observation is robust across a broad range of parameter values, as shown in S2 Text. Consequently, for InIs, higher concentrations are required to prevent infection than suggested after conducting the respective *in vitro* experiments. For CRAs, predictions are parameter dependent, S2 Text. Rearranging Eqs (25)–(27) allows to directly compute the drug concentration that prevents infection with *x* percent probability (the EC_*x*_) from the corresponding *in vitro* 50% inhibitory concentration IC_50_ (derivations in S3 Text): In case of exposure *to a single virus particle* we get

$$\begin{array}{c} {\text{EC}}_{x}={\text{IC}}_{50}\cdot (F\cdot \frac{x}{100\cdot C-x}{)}^{1/m}, \end{array}$$ (28)

where EC_*x*_ is the drug concentration that achieves *x* percent of prophylactic efficacy and the term *F* ≥ 1 is a drug class specific factor

$$\begin{array}{c} F=\{\begin{array}{cc} (\text{CL}+\frac{\beta \cdot T_{u}}{\rho })/\text{CL}, & \text{for}\;\text{CRA}\\ 1, & \text{for}\;\text{RTI}\\ 1/\vartheta , & \text{for}\;\text{InI}. \end{array} \end{array}$$ (29)

and

$$\begin{array}{c} C≔\frac{R_{0}\left(⌀\right)}{R_{0}\left(⌀\right)-1}\approx 1, \end{array}$$ (30)

if *R*_0_(⌀) ≫ 1. Importantly, when exposure to multiple viruses occurs, the concentration-prophylaxis profile is no longer an Emax equation for any inhibitor class, Fig 3A–3C. Furthermore, the slope parameter increases and the EC_50_ may exceed the *in vitro* measurable IC_50_ value. At large inoculum, the corresponding profiles become switch-like. For protease inhibitors (PIs), we derive a power function to describe their prophylactic efficacy (mechanistic derivation in S1 Text):

$$\begin{array}{ccc} \varphi \left(\hat{V}\right)=\frac{1}{R_{0}\left(⌀\right)-1}\cdot \frac{D^{m}}{{\text{IC}}_{50}^{m}} & =C\cdot \frac{D^{m}}{{\text{IC}}_{50}^{m}} & \;\text{(PI)}\;\;\;\text{for}\;0\leq \varphi \leq 1, \end{array}$$ (31)

where *C* << 1 is a constant. Moreover, for realistic (large) *R*_0_(⌀) ≫ 3 their plasma concentration has to exceed their IC_50_ to decrease the probability of infection by at least 50%, Fig 3D. Similarly, we can rearrange the equation above and obtain

$${\text{EC}}_{x}={\text{IC}}_{\text{50}}\cdot {\left(G\cdot \frac{x}{100}\right)}^{1/m},$$ (32)

for the exposure to a *to a single virus*, where *G* ≔ *R*_0_(⌀) − 1. Again, in case of exposure to multiple viruses, the slope parameter and EC_50_ increase, making the prophylactic efficacy of PIs exhibiting a switch-like behaviour as can be seen in Fig 3D. This switch-like behaviour makes the prophylactic use of PIs vulnerable to non-adherence, as well as general variations in concentrations (e.g. pharmacokinetics, inter-individual variability), and the prophylactic efficacy with these inhibitors may alternate between zero- or complete protection.

### Prophylactic efficacy of treatment-approved antivirals

The combination of the nucleoside reverse transcriptase inhibitors (NRTIs) emtricitabine and tenofovir (Truvada) is the only intervention approved for pre-exposure prophylaxis (PrEP). According to our previous estimates [18], Truvada provides 96% protection in fully adherent individuals, which is in line with clinical estimates of 86-100% protection in the IPERGAY study [1], 58-96% in the PROUD study [2] and 96% in the Partners PrEP OLE study in apparently highly adherent individuals. The VOICE [3] and FEM-PrEP [52] studies indicated that Truvada may not prevent infection in poorly adherent individuals.

Currently, a number of drugs are under investigation for PrEP repurposing [6]. Notably, all currently investigated compounds are patent-protected and may not be affordable in resource-constrained countries hit hardest by the epidemic. In this work, we wanted to unselectively assess the utility of treatment-approved antivirals for prophylaxis and to assess whether currently neglected (patent-expired) compounds may be cost-efficient alternatives to be further explored in non-profit prophylaxis programmes.

**Fig 4:**
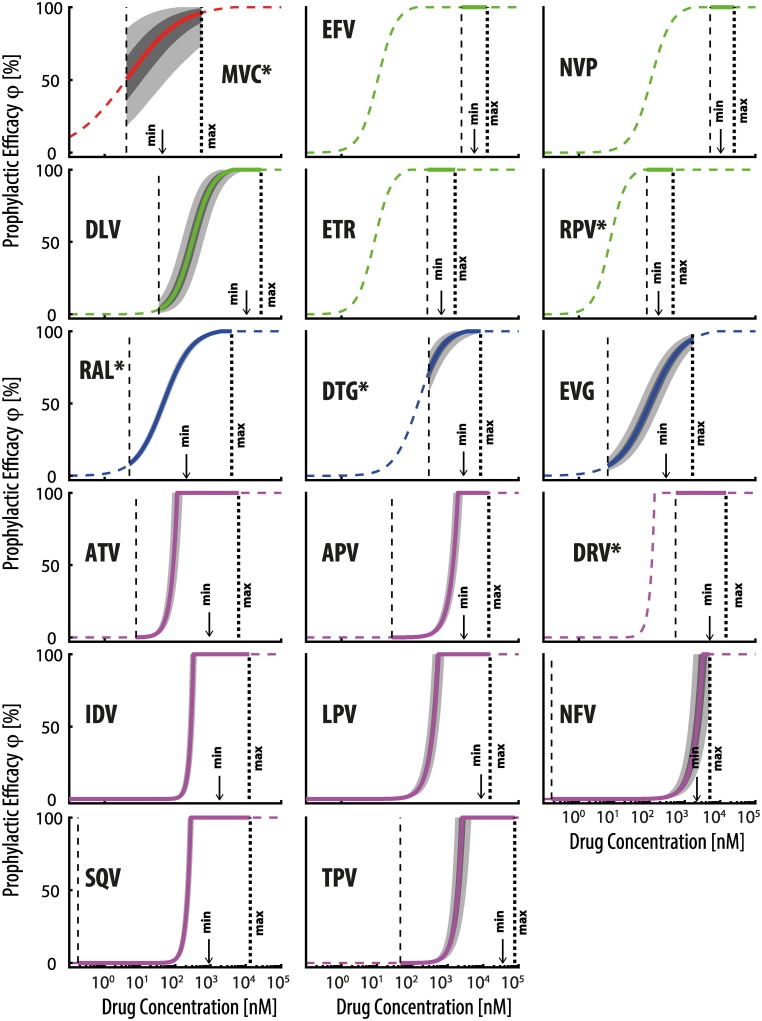
Drug specific prophylactic efficacy. Solid and dashed colored lines depict the concentration-prophylaxis profile for the individual drugs. The solid lines represent the concentration-prophylaxis profiles and light and dark grey areas indicate the quartile ranges and 5-95% ranges of the concentration-prophylaxis profile, considering uncertainty in pharmacodynamic parameters (Table 2) and the distribution of viral inoculum sizes after homosexual exposure to HIV using the virus exposure model’ (Methods section and [18]). Maximum clinically achievable concentrations *C*_max_ for chronic oral administration of the standard dosing regimen and a lower bound concentration *C*_low_ that would be achieved if the last dose had been taken three days prior to virus exposure are marked by thick and thin vertical black dashed lines respectively. For IDV, LPV, NFV and SQV *C*_low_ falls below the range of the x-axis. Downward pointing arrows indicate minimum (pre-dose) concentrations achieved for standard regimen in *adherent* individuals as reported in [40], [96] and [95]. MVC -maraviroc, EFV -efavirenz, NVP -nevirapine, DLV -delavirdine, ETR -etravirine, RPV -rilpivirine, RAL -raltegravir, EVG -elvitegravir, DTG -dolutegravir, ATV -atazanavir, APV -amprenavir, DRV -darunavir, IDV -indinavir, LPV -lopinavir, NFV -nelfinavir, SQV -saquinavir, TPV -tipranavir. *recently or currently tested for PrEP.

We utilized comprehensive sets of drug-specific pharmacodynamic- and pharmacokinetic parameters (Table 2) to parameterize Eq (10) and to predict the prophylactic efficacy of treatment approved CRAs, non-nucleoside reverse transcriptase inhibitors (NNRTIs), InIs and PIs at clinically relevant concentration ranges (the class of NRTIs have been analyzed in earlier work [18]). Moreover, we sampled the extent of viral exposure (number of viruses transmitted and reaching a replication-enabling compartment; Eq (22)) from a previously parameterized distribution [18] that accurately reflects transmitter virus loads and drug-free infection probabilities after sexual contact. The resultant benchmark is depicted in Fig 4. Fig 4 allows for an initial screen of the utility of the various drugs for oral PrEP. Most analyzed drugs, except for maraviroc (MVC), raltegravir (RAL), elvitegravir (EVG) and nelfinavir (NFV), potently prevent infection at concentrations ranges typically encountered in fully adherent individuals during treatment (range between minimum- to maximum concentration, [*C*_min_; *C*_max_]). During prophylaxis, adherence to the dosing regimen is a major problem and we thus consider a lower bound concentration that would arise if the drug had not been taken for three days prior to exposure *C*_low_ (thin dashed vertical line in Fig 4) to emphasise a ‘pharmacokinetic safety margin’ in case of poor adherence. Numerical values for the computed maximum prophylactic efficacy and the efficacy at the lower bound concentrations are reported in Table 3 alongside with estimated EC_50_ and EC_90_ values. While in Table 3 we report the EC_50_ and EC_90_ values after challenge with a single virus $\hat{V}$, the corresponding values after virus challenges sampled from the distribution for homosexual exposure Eq (24) were almost identical, see S4 Text for a comparison. Our simulations indicate a residual risk of infection for most analyzed drugs. Notably, most protease inhibitors may confer anything from none- to absolute protection within relevant concentration ranges, [*C*_low_; *C*_max_], which highlights a severe limitation to their PrEP use in the context of poor adherence or pharmacokinetic (intra-/inter individual) variability. An exception among this rule is darunavir (DRV), which is predicted to be almost fully protective for the entire concentration range.

**Table 3:** Prophylactic efficacy and sensitivity to incomplete adherence. The table shows the prophylactic efficacy (% reduction in infection probability) of all investigated drugs at their respective maximum achievable drug concentrations after chronic oral administration of the standard regimen and its efficacy at a concentration level that would be reached if the last dose had been taken least three days prior to virus exposure $C_{low}=C_{min}\cdot e^{-2\cdot 24\cdot k_{e}}$, with *k*_*e*_ = ln(2)/*t*_1/2_ and halflifes *t*_1/2_ reported in Table 2. The 5-95% range of these estimates are shown in brackets and consider uncertainty in pharmacodynamic parameters IC_50_, *m* and variability in virus exposure after homosexual contact, according to the ‘virus exposure model’ (Methods section and Duwal et al. [18]. The last two columns show the EC_50_ and EC_90_ in the case when an individual was exposed to a single virus $\hat{V}$. MVC -maraviroc, EFV -efavirenz, NVP -nevirapine, DLV -delavirdine, ETR -etravirine, RPV -rilpivirine, RAL -raltegravir, EVG -elvitegravir, DTG -dolutegravir, ATV -atazanavir, APV -amprenavir, DRV -darunavir, IDV -indinavir, LPV -lopinavir, NFV -nelfinavir, SQV -saquinavir, TPV -tipranavir. *currently investigated for PrEP.

	prophylactic efficacy *φ* [%]				${EC}_{50}(\hat{V})$	$EC_{90}(\hat{V})$
drug	*φ*(*C*_max_)		*φ*(*C*_low_)		[nM]	[nM]
MVC*	96.10	(74.11;100)	50.12	(18.63;85.42)	11.45	349.63
EFV	100	(100;100)	100	(100;100)	10.55	36.23
NVP	100	(100;100)	100	(100;100)	114.06	438.06
DLV	100	(100;100)	3.38	(0.88;10.19)	329.50	1254.58
ETR	100	(100;100)	100	(100;100)	8.45	26.75
RPV*	100	(100;100)	100	(99.02;100)	7.61	22.55
RAL*	100	(100;100)	8.15	(6.32;10.23)	45.40	302.36
DTG*	100	(99.03;100)	72.12	(57.77;84.85)	145.18	722.23
EVG	94.61	(89.02;97.97)	6.96	(3.66;12.49)	108.66	976.25
ATV	100	(100;100)	0.08	(0.04;0.15)	87.44	108.79
APV	100	(100;100)	0.01	(0.01;0.03)	1394.96	1848
DRV*	100	(100;100)	100	(100;100)	118.32	139.24
IDV	100	(100;100)	0	(0;0)	280.80	319.71
LPV	100	(100;100)	0	(0;0)	389.69	519.09
NFV	100	(64.01;100)	0	(0;0)	2253.66	3118.34
SQV	100	(100;100)	0	(0;0)	227.29	266.66
TPV	100	(100;100)	0	(0;0.02)	1944.89	2458.09

Of the analyzed non-PI drugs, the NNRTIs efavirenz (EFV), nevirapine (NVP), etravirine (ETR) and rilpivirine (RPV) are extremely potent with regard to prophylaxis: These drugs prevent infection, even when the drug had not been taken for three consecutive days, Table 3. Notably, NVP and EFV are patent-expired and may represent suitable candidates for use in resource-constrained settings (price per day ≈ 0.1US$). The co-receptor antagonist maraviroc (MVC) and the integrase inhibitor dolutegravir (DTG) retain some prophylactic efficacy (50 and 72% respectively) at lower bound concentrations *C*_low_. The CRA maraviroc (MVC), the NNRTI rilpivirine (RPV) and the InI raltegravir (RAL) are currently investigated for use as PrEP compounds (long-acting injections of RPV and RAL; oral- or topical application of MVC). In our simulations the predicted PrEP efficacy of these drugs would drop to 8% (RAL) and 50% (MVC) when the drug had not been taken for three consecutive days prior to virus exposure. Notably, RPV remained 100% effective.

Lastly, we want to note that our predictions are based on viral dynamics parameters that may under-predict prophylactic efficacy, as indicated in S2 Text. The main purpose of this modelling study was to rule out drug candidates, based on lack-off- or uncertain- prophylactic efficacy. While some drugs’ prophylactic efficacy might be under-predicted, this *conservative* choice of parameters provides a more solid scientific basis for the remaining candidates that are predicted to be potent.

## Discussion

Our intent was to develop a tool to screen out unsuitable candidates for PrEP based on unfavourable pharmacokinetic and pharmacodynamic characteristics. Clearly, the attributes which make any compound favourable extend beyond PK/PD, and critically also depend on tolerability, ease of dosing, cost and acceptability. Nevertheless, screening antiretroviral agents based on their intrinsic antiviral activity, mode of action, duration of efficacy beyond the dosing interval, and tolerance for missed dosing is a logical starting point when assessing potential candidates for PrEP.

Strikingly, we observed that *in vitro* measured drug potency may over-estimate PrEP potency in a drug-class specific manner. For all non-RTI drugs dosing schedules in clinical trials may have to be adjusted accordingly to reach the desired prophylaxis endpoints (% protection). We provide an easy-to-use software tool to determine the corresponding target concentrations (www.systems-pharmacology.org/prep-predictor).

For non-PI drugs, we observed a more graded relationship between their prophylactic efficacy and drug concentrations. At low virus inoculum sizes, the slope of their concentration-prophylaxis profile is largely determined by the slope coefficient that describes their direct effects [40]. Notably, for PIs we observed a very steep concentration prophylaxis profile, suggesting that within clinically relevant ranges for oral PrEP (Fig 4) their efficacy is likely to switch between zero- and complete protection, in an ‘either-or’ scenario. This characteristic renders PIs particularly vulnerable to poor adherence and drug-drug interactions. An intuitive explanation for this steep concentration-prophylaxis profile of PIs (power function in Eq (31)) is based on its unfortunate drug-to-target stoichiometry: A single late infected cell T_2_ produces hundreds of infectious viruses on average (using parameters from Table 1 *a*_6_/*a*_3_ = 670) and a PI needs to prevent all of them from becoming infectious to fully prevent infection. By contrast, all other compounds only need to prevent a single viral entity from progressing, explaining the proportionality to the EMAX equation seen in Eqs (25)–(27).

By screening all treatment-approved antivirals for their PrEP utility, we predicted that efavirenz (EFV), nevirapine (NVP), etravirine (ETR), rilpivirine (RPV) and darunavir (DRV) may fully prevent infection after oral application and in case of poor adherence (Table 3 and Fig 4). Notably, these compounds have favourable inhibitory quotients (clinically achieved concentrations vastly exceed their EC_50_) and their long elimination half-lives guarantees that inhibitory quotients stay in that favourable range. The drugs maraviroc (MVC) and dolutegravir (DTG) potently prevent infection but may allow for HIV transmission when individuals poorly adhere to the medication. Notably, the NNRTIs EFV, NVP, RPV and ETR exhibit long elimination half-lives (30-40h) and achieve concentrations required for PrEP to act quickly, and durably. However, there are some safety concerns with liver toxicity, which contraindicate e.g. the use of NVP in uninfected individuals. Liver toxicity to ETR remains to be elucidated in the context of prophylaxis. Skin reactions (ETR and EFV) and neuropsychiatric effects (EFV) have been reported in the context of HIV treatment that need to be evaluated in the context of potential PrEP applications. Likewise, skin reactions and rare liver toxicities with DRV need careful assessment in the context of PrEP repurposing. Moreover, the particular concentration-prophylaxis profile, as depicted in Fig 4, argues for a form of DRV administration that is not dependent on daily dosing for maintaining drug levels (e.g. slow release or nanoparticle formulations). For rilpivirine (RPV), our simulations suggest that near complete protection can be achieved when concentrations exceed EC_90_, Fig 4. RPV is currently investigated as a long-acting formulation in HPTN076 using 1200mg injections every 2 month which yields tough concentrations (median 186 nM) well in excess of this target. However, significant variability is still observed related to gender, and between injections on different occasions [51] which could be incorporated into future model generations. Besides rilpivirine, maraviroc (MVC; 300mg once daily), and raltegravir (RAL) are currently clinically investigated for oral PrEP. Our simulations suggest MVC may incompletely prevent infection even at maximum concentrations and that its efficacy steadily drops with declining levels down to 50% when the drug had not been taken for three days prior to exposure. Results from the NEXT-PrEP (HPTN 069) phase II study observed that MVC may not be potent enough on its own and that among those acquiring HIV infection, MVC concentrations were low, absent or variable [53]. Our model prediction is consistent with the reported lack of efficacy of MVC as PrEP in animals and human explant samples [54] and suggests that the potency of MVC, against infection may be less than its potency in preventing HIV replication (EC_50_ > IC_50_, EC_90_ > IC_90_). However, EC_50_, EC_90_ estimates for co-receptor antagonists are highly parameter sensitive (S2 Text) warranting further research into elucidating the early infection dynamics. Using the parameters presented in Table 1, we estimate that the EC_90_ may be around 350nM, which is approximately 70 times larger than its IC_50_ (conversion formula provided in the results section). Notably, during the dose finding for MVC an IC_90_ of only 3.9nM (2ng/ml) was considered and this estimate was taken directly to determine target concentrations providing 90% prophylactic efficacy. Other compounds currently under investigation (HPTN-083) [55], but not evaluated in our study are the novel long-acting integrase inhibitor cabotegravir.

Our model has several limitations, but also a number of important advantages. Our simulations do not take into account drug concentrations at the site of mucosal exposure (e.g. cervix, rectum) [51, 56]. These concentrations have, however, not been validated as targets for successful prevention or treatment, whereas data exist (albeit limited) for plasma drug concentrations. Instead, we modelled based on *unbound* concentrations, in line with the broadly accepted ‘free drug hypothesis’. Under the ‘free drug hypothesis’, the unbound concentrations are assumed to be available at the target site to exert pharmacological effects. For drugs highly bound to plasma protein (> 90%), naturally, their *total* concentrations at sites other than the plasma are magnitudes lower [56]. Strikingly, however, the unbound concentrations are identical [57]. Therefore, throughout the work, we assumed, according to the ‘free drug hypothesis’ [58] that the unbound concentrations in plasma and at the target site coincide, where the latter exerts the antiviral effect [59, 60]. All analyzed NNRTIs, InIs and PIs, except for raltegravir (RAL), are highly lipophilic, enabling the *unbound* drug to rapidly cross cellular membranes, generating an equilibrium between the *unbound* drug on either side of the cellular membrane [61]. Even for the weakly lipophilic compound raltegravir, intracellular concentrations are proportional to plasma concentrations by a factor precisely resembling their unbound moiety [62, 63], strongly arguing for the validity of the ‘free drug hypothesis’ for all analyzed drugs. However, ultimate proof in terms of local measurements in humans are lacking currently and may be difficult to obtain experimentally. On the contrary, nucleoside reverse transcriptase inhibitors (NRTIs), which we analysed in a previous work [18] are not expected to obey the ‘free drug hypothesis’ [17, 30, 64]. These compounds need to be actively taken up by cells and converted intracellularly into pharmacologically active triphosphates (NRTI-TP). Since the expression of transporters and intracellular enzymes is likely cell-specific, different cell types may contain vastly different concentrations of pharmacologically active compound. It is therefore entirely unclear what relevance concentration measurements of NRTI-TPs in tissue homogenates [65] (containing HIV target- and non-target cells) from sites of viral exposure (e.g. cervix, rectum) have in terms of prophylaxis.

Utilising the virus exposure model Eqs (23) and (24), we estimated the probability of virus clearance (and the prophylactic efficacy *φ*) as a function of the number of viruses ultimately reaching a target cell environment, and not as a function of mucosal exposure. The quantitative role of a number of physiological processes underlying primary infection is currently not fully resolved and impossible to measure in humans (e.g. the cells involved at the local site of exposure, their abundance, locations, their capabilities to transduce virus through physiological barriers and the respective *R*_0_s). It is known however, that the virus has to overcome a number of physiological bottlenecks/barriers to reach a compartment that permits viral expansion. Despite the mucosal barrier, the sub-mucosal target cell density might initially be low [66], such that only a tiny fraction of viruses find a target cell before being cleared. It has also been reported [66–68], that target cells are subsequently recruited to the site of initial exposure due to inflammation and seminal exposure, mitigating the ‘low target cell bottleneck’ subsequently. If the low target-cell bottleneck is only prevalent during the first replication cycle it can also be modelled by simply considering a smaller virus inoculum that reaches a target-cell environment. In our approach, to obviate model- and parameter uncertainties, we chose a minimal/parsimoneous, data-driven approach that treats all physiological barriers as a single bottleneck lumped in terms of the ‘success probability’ *r* in Eq (24).

The target cell environment herein is a compartment that is decisive for establishing- and shedding infection (this compartment requires *R*_0_ > 1). We also assumed that this compartment is well-perfused at the time scale of interest. Under this assumption, viral kinetic parameters measured in plasma coincide with kinetic parameters at the target cell environment, after converting the deterministic reaction parameters to their respective stochastic counterparts (Table 1).

Notably, the model (see Methods section) is calibrated [18] to reflect the per-contact infection risks for typical transmitter virus loads and different modes of sexual exposure (homo- and heterosexual), but can also be adapted to model intravenous exposure by e.g. injection. The calibrated virus exposure model [18] (see Methods section) predicts that either none- or a single infectious virus enter a replication enabling compartment in the majority of hetero-/homosexual contacts. Thus, we suggest that ${EC}_{50}(\hat{V})$, ${EC}_{90}(\hat{V})$ values stated in Table 3 provide a good proxy for the drug-specific prophylactic potency after sexual exposure to HIV (see also S4 Text for a comparison). Importantly, we also observed that increasing the inoculum size decreases the prophylactic efficacy of all drug classes considered (as suggested by increasing EC_50_ and EC_90_) and increases the steepness of the concentration prophylaxis profile, Fig 3. PIs in particular displayed an almost switch-like prophylactic profile in the case of large inoculum sizes. These observations strikingly indicate that preventive target concentrations can depend on the route of transmission. I.e., intravenous exposure to HIV (larger inoculum sizes compared to sexual exposure) may require higher concentrations for HIV prevention.

When *R*_0_(⌀) is relatively large, we find that our predictions of prophylactic efficacy and -potency for or CRAs, RTIs and InIs are relatively invariant to parameter changes (compare Eqs (25)–(30)). However, we find that when considering an extremely broad range of 1.7 < *R*_0_(⌀) < 112 values, as in S2 Text, that the parameters used are rather *conservative* in the sense that they disfavour the drugs and may under-predict prophylactic efficacy. With regard to our work’s aim (screen out candidates based on lack-off-, or uncertain potency) such a *conservative* parameter choice should be preferred. For PIs, although highly sensitive to changes *R*_0_ (compare Eqs (31) and (32)) the qualitative statements made (the prophylactic potency is less than suggested by IC50, as well as the steep shape of the concentration-prophylaxis profile) are unaffected for arbitrary, yet realistic parameters, as analysed in S2 Text. However, in the provided software tool (www.systems-pharmacology.org/prep-predictor) it is possible to freely change all virus dynamics parameters. Notably, Ribero et al. [69] have recently estimated *R*_0_ ≈ 8 during *acute* infection (≤ 10 days after exposure, virus is detectable), which is much lower than the value used by us *R*_0_ ≈ 67, which considers viral replication immediately after exposure, in the so-called *eclipse phase* before virus becomes detectable. Our *R*_0_ value is relatively high because we assume a lower CL (clearance of free virus) during this early phase of infection, in line with other modelling approaches [14, 33] and in line with the observation that adaptive immune responses develop only after about 14 days post exposure [70]. However, if we utilise CL = 23 (1/day), as in Ribero et al. [69], we obtain similar values of *R*_0_.

The knowledge of concentration-prophylaxis relationships between drug classes, and for each component of a particular drug class allows for the intelligent design of PrEP regimens, including how quickly protection can be achieved after a loading dose and how forgiving the regimen is towards missed dosing events. In related article [27] we develop a sophisticated simulation framework that allows to make use of population pharmacokinetic models, to fully explore inter-individual pharmacokinetics and to assess sensitivity towards dosing, individual pharmacokinetic variability and timing of viral challenges.

Our model can be adapted or developed in a number of ways. On a technical side, the analytical solutions provided in the article can be neatly integrated into hybrid stochastic-deterministic algorithms that consider time-varying drug concentrations (pharmacokinetics), as outlined in an accompanying article [27]. In brief, therein we utilize analytic solutions for the extinction probability, Eq (22), to define a set of states where extinction is feasible (*extinction simplex*). Whenever trajectories leave the *extinction simplex*, simulations can be stopped and a hybrid stochastic-deterministic trajectory can be safely classified as an *infection event*. Regarding applications, the separate impact of treatment as prevention [71] (in the case of the donor) versus prophylactic efficacy in the exposed individual can be readily simulated by calibrating the virus load distribution in potential transmitter populations (see ‘exposure model’ in the Methods section). The effect of PrEP on the transmission of resistance can be estimated by altering *R*_0_(⌀) (the fitness cost of resistance) and by simultaneously increasing IC_50_ in Eq (10) (extent of resistance). The fitness cost of resistance translates into a decreased transmissibility of resistance in the absence of drugs (Eqs (19)–(21)), while the extent of resistance translates into an increased transmissibility relative to the wildtype at increasing drug concentrations, as e.g. illustrated in [18] (Figure 3 therein). Consequently, provided any transmitted resistance confers some fitness defect, prophylaxis may increase the frequency of transmitted resistance relative to the wildtype, but not its absolute occurrence [18, 72]. Since resistance to HIV drugs generally develops in a stepwise manner, the change in EC_50_ following acquisition of a resistance mutation can be introduced into this model, to identify a zone of selective pressure for the *de novo* evolution or spread of resistance under PrEP. However, during the early events when the virus infection can still be averted, the population size is too small for resistance to appear *de novo*: A single point mutation appears with probability 1 − (1 − *μ*)^k^ at a particular base, where *μ* ≈ 2.2 ⋅ 10^-5^ is the per base mutation rate of HIV during reverse transcription [73] and *k* is the number of reverse transcription (= cell infection) events. Thus, *de novo* resistance can be assumed to appear, if e.g. PrEP had not been taken at the time of exposure, such that the infection expanded exponentially, and when PrEP is (re-)initiated some time after this early infection has been established. *De novo* resistance development in the context of poor adherence can be modelled in analogy to the work conducted by Rosenbloom et al. [74].

It is well known that the establishment of a latent reservoir is the major barrier to viral extinction during treatment [75] and this reservoir may be established as early as 3 days post infection [76, 77]. In the current framework, we computed viral extinction when *t* → ∞, assuming drug concentrations stayed constant. Thus, extinction estimates are not affected by the inclusion of a long lived cellular compartment. In an accompanying article [27] we overcome this assumption, explicitly considering drug pharmacokinetics and e.g. short-course prophylaxis. In the accompanying article infection of long lived cells are considered as an algorithmic stopping criterium: I.e., whenever long lived cells become infected, viral extinction is considered infeasible.

In summary, we have developed a mechanistic modelling tool to *a priori* screen antivirals for their prophylactic utility. Our approach revealed that *in vitro* measured drug potency (IC_50_, IC_90_) should not be used directly to identify lower bound effective concentrations in PrEP trials: With the exception of reverse transcriptase inhibitors, PrEP potency may be less than *in vitro* drug potency, i.e. higher concentrations of drug are required for prophylaxis than suggested by their *in vitro* potency. Consequently, when clinical trial design is guided by *in vitro* drug potency, prophylactic dosing regimen may be selected that attain insufficient concentrations to adequately prevent HIV infection.

Instead, we recommend to use the tool provided (www.systems-pharmacology.org/prep-predictor) to translate *in vitro* drug potency into prophylactic efficacy. We used the developed methods to assess the prophylactic utility of all treatment approved antivirals, allowing to rule out particular candidates by lack-of-, or uncertain prophylactic efficacy. To this end, we presented results using viral dynamics parameters that may under-predict prophylactic efficacy (S2 Text). These preliminary screens indicated that darunavir (DRV), efavirenz (EFV), nevirapine (NVP), etravirine (ETR) and rilpivirine (RPV) may fully prevent infection at concentrations typically achieved during treatment and with an adequate ‘pharmacokinetic margin’. Notably, this prediction is robust across a wide range of (uncertain) parameters (S2 Text). Moreover, we predicted that maraviroc (MVC) and dolutegravir (DTG) can potently prevent infection, but that these drugs do not provide a comparable ‘pharmacokinetic margin’. Furthermore, predictions for MVC are uncertain with respect to viral dynamics parameters (efficacy may both be over- or underpredicted). A next logical step is to further trim this candidate set by ruling out compounds with ominous safety profiles, followed by an assessment of different dosing (roll-out) schemes.

## Supporting information

S1 Text The Supplementary Text contains a step-by-step derivation of the equations for the extinction/infection probability presented in the main article. (PDF)

S2 Text The Supplementary Text contains a sensitivity analysis with respect to viral dynamics parameters. (PDF)

S3 Text The Supplementary Text contains details of the IC_50_-to-EC_50_ conversion. (PDF)

S4 TextThe Supplementary Text compares the EC_50_ and EC_90_ (antiviral concentrations that provide 50- and 90% protection) after viral challenge with (i) a single virus $\hat{V}$, and after virus challenges (ii) sampled from the distribution for homosexual exposure, Eq (24).(PDF)

S5 Text The Supplementary Text contains details of the protein-binding adjustment for pharmacodynamic parameters. (PDF)
