# Supplementary material for: Mechanistic framework predicts drug-class specific utility of antiretrovirals for HIV prophylaxis
Source: PLoS Comput Biol. 2019 Jan 30;15(1):e1006740. doi: 10.1371/journal.pcbi.1006740 (PMC6370240; doi:10.1371/journal.pcbi.1006740)
Supplement: S1 Text — (PDF) [file pcbi.1006740.s001.pdf]

Supplementary Text S1.

S1.1 Derivation of extinction probability

For the ease of notation we will introduce the unit vectors $\widehat{\mathrm{V}}$, $\widehat{\mathrm{T}}_1$ and $\widehat{\mathrm{T}}_2$ which represent the states where only one infected compartment is present (either virus, early or late infected cells)

$$\widehat{\mathrm{V}} = \begin{bmatrix}1\\0\\0\end{bmatrix}, \ \widehat{\mathrm{T}}_1 = \begin{bmatrix}0\\1\\0\end{bmatrix}, \ \widehat{\mathrm{T}}_2 = \begin{bmatrix}0\\0\\1\end{bmatrix} \tag{S1.1}$$

Any state of the system can be expressed as a linear combination of the unit vectors above, e.g. $5 \cdot \widehat{\mathrm{V}} \oplus 3 \cdot \widehat{\mathrm{T}}_1 \oplus 12 \cdot \widehat{\mathrm{T}}_2$ denotes the state where we have 5 viruses, 3 early infected cells and 12 late infected cells.

One-step transitions/embedded Markov Chain

The viral replication cycle (Fig.1, main article) can be interpreted as Markov jump processes. Since we are interested in the eventual clearance of virus ($t \to \infty$) it is useful to construct its embedded Markov chain [1]. Of particular interest are the *one-step* transition probabilities i.e, the probability of advancing from the current state to a next, conditioned on leaving the current state. Note that the transition probabilities are time-invariant under the assumption of constant drug effects as outlined in the main article.

Without a loss of generality, Y_0 and Y_1 denote the initial and the next state respectively.

A virus can be cleared by immune system or due to an unsuccessful infection attempt. The probability of this event/path $\widehat{\mathrm{V}} \to *$ is given by

$$\mathbb{P}(Y_1 = \mathbf{0} | Y_0 = \widehat{\mathrm{V}}) = \frac{a_1(D)}{a_1(D) + a_4(D)} = \varrho. \tag{S1.2}$$

where (D) indicates that the propensity $a_{(\cdot)}$ may be a function of some inhibitor concentration, as outlined in eqs.(11)–(16) (main manuscript) and $\mathbf{0}$ denotes the extinction state $[0, 0, 0]^T$.

Similarly, the probability of the path $(\widehat{\mathrm{V}} \to \widehat{\mathrm{T}}_1)$ is given by:

$$\mathbb{P}(Y_1 = \widehat{\mathrm{T}}_1 | Y_0 = \widehat{\mathrm{V}}) = \frac{a_4(D)}{a_1(D) + a_4(D)} = \zeta. \tag{S1.3}$$

In analogy we have for all other *one step* paths

$$\mathbb{P}(Y_1 = \mathbf{0} | Y_0 = \widehat{\mathrm{T}}_1) = \frac{a_2}{a_2 + a_5(D)} = \vartheta\,, \qquad \mathbb{P}(Y_1 = \widehat{\mathrm{T}}_2 | Y_0 = \widehat{\mathrm{T}}_1) = \tfrac{a_5(D)}{a_2 + a_5(D)} = \xi \tag{S1.4}$$

$$\mathbb{P}(Y_1 = \mathbf{0} | Y_0 = \widehat{\mathrm{T}}_2) = \frac{a_3}{a_3 + a_6(D)} = \chi\,, \qquad \mathbb{P}(Y_1 = \widehat{\mathrm{V}} + \widehat{\mathrm{T}}_2 | Y_0 = \widehat{\mathrm{T}}_2) = \tfrac{a_6(D)}{a_3 + a_6(D)} = \gamma \tag{S1.5}$$

where eqs. (S1.2)–(S1.5) define the entries of the transition matrix of the embedded Markov chain.

Consequently we have $\varrho + \zeta = 1$, $\vartheta + \xi = 1$ and $\gamma + \chi = 1$. From the drug-class specific direct effects outlined in eqs.(11)–(16) (main article), it is evident that RTIs and CRAs affect the transition probability ϱ and ζ. Similarly, InIs affect ϑ and ξ and PIs affect γ and χ. For simplicity we skipped (D) in the short notations.

Note that all paths of length one (one step transitions) irrespective of the number of reaction educts can be expressed by eqs. (S1.2)–(S1.5). E.g. the probability of the path $\begin{bmatrix}n\\0\\1\end{bmatrix} \to \begin{bmatrix}n+1\\0\\1\end{bmatrix}$ is given by

$$\mathbb{P}(Y_1 = (n+1) \cdot \widehat{\mathrm{V}} \oplus \widehat{\mathrm{T}}_2 \mid Y_0 = n \cdot \widehat{\mathrm{V}} \oplus \widehat{\mathrm{T}}_2) \quad = \quad \frac{a_6(D)}{a_3 + a_6(D)} = \gamma \tag{S1.6}$$

where $n \in \mathbb{Z}^+$. The probability of an arbitrary (multi-step) path, e.g. $\widehat{\mathrm{V}} \to \widehat{\mathrm{T}}_1 \to \widehat{\mathrm{T}}_2$ can be computed from the single step transition probabilities, eqs. (S1.2)–(S1.5) applying the fundamental laws of probability

$$\mathbb{P}(Y_2 = \widehat{\mathrm{T}}_2 | Y_0 = \widehat{\mathrm{V}}) = \mathbb{P}(Y_1 = \widehat{\mathrm{T}}_1 | Y_0 = \widehat{\mathrm{V}}) \cdot \mathbb{P}(Y_2 = \widehat{\mathrm{T}}_2 | Y_1 = \widehat{\mathrm{T}}_1) = \zeta \cdot \xi \tag{S1.7}$$

Likewise, if multiple paths can lead to the state of interest, the probability is computed by summing over all possible paths.

In that respect, let us consider the probability that the virus goes extinct in the first replication cycle $\mathbb{P}(Y_r = \mathbf{0}|Y_0 = \widehat{\mathrm{V}})$, i.e. starting from a single virus, we consider the paths $\widehat{\mathrm{V}} \to \mathbf{0}$, $\widehat{\mathrm{V}} \to \widehat{\mathrm{T}}_1 \to \mathbf{0}$ and $\widehat{\mathrm{V}} \to \widehat{\mathrm{T}}_1 \to \widehat{\mathrm{T}}_2 \to \mathbf{0}$ which is

$$\begin{aligned} \mathbb{P}(Y_r = \mathbf{0}|Y_0 = \widehat{\mathrm{V}}) &= \mathbb{P}(Y_1 = \mathbf{0}|Y_0 = \widehat{\mathrm{V}}) \\ &+ \mathbb{P}(Y_2 = \mathbf{0}|Y_1 = \widehat{\mathrm{T}}_1) \cdot \mathbb{P}(Y_1 = \widehat{\mathrm{T}}_1|Y_0 = \widehat{\mathrm{V}}) \\ &+ \mathbb{P}(Y_3 = \mathbf{0}|Y_2 = \widehat{\mathrm{T}}_2) \cdot \mathbb{P}(Y_2 = \widehat{\mathrm{T}}_2|Y_1 = \widehat{\mathrm{T}}_1) \cdot \mathbb{P}(Y_1 = \widehat{\mathrm{T}}_1|Y_0 = \widehat{\mathrm{V}}). \end{aligned} \quad (S1.8)$$

Or, using the short notation

$$\mathbb{P}(Y_r = \mathbf{0}|Y_0 = \widehat{\mathrm{V}}) = \varrho + \zeta \cdot \vartheta + \zeta \cdot \xi \cdot \chi \quad (S1.9)$$

Complementarily, we can compute the probability that $n > 0$ progeny viruses are produced in the first replication cycle, i.e. starting from a single virus, all paths that reach the late infected T-cell stage $\widehat{\mathrm{V}} \to \widehat{\mathrm{T}}_1 \to \widehat{\mathrm{T}}_2$, then fire reaction (9) (main article; virus release) n times before finally reaction (6) (main article; clearance of late infected T-cell) occurs.

$$\begin{aligned} \mathbb{P}(Y_r = n \cdot \widehat{\mathrm{V}}|Y_0 = \widehat{\mathrm{V}}) &= \mathbb{P}(Y_1 = \widehat{\mathrm{T}}_1|Y_0 = \widehat{\mathrm{V}}) \times \\ &\quad \mathbb{P}(Y_2 = \widehat{\mathrm{T}}_2|Y_1 = \widehat{\mathrm{T}}_1) \times \\ &\quad \prod_{i=0}^{n-1} \mathbb{P}(Y_{3+i} = \widehat{\mathrm{T}}_2 + (i+1) \cdot \widehat{\mathrm{V}} \mid Y_{2+i} = \widehat{\mathrm{T}}_2 + i \cdot \widehat{\mathrm{V}}) \times \\ &\quad \mathbb{P}(Y_{n+3} = n \cdot \widehat{\mathrm{V}}|Y_{n+2} = \widehat{\mathrm{T}}_2 + n \cdot \widehat{\mathrm{V}}). \end{aligned} \quad (S1.10)$$

Or in short notation

$$\mathbb{P}(Y_r = n \cdot \widehat{\mathrm{V}}|Y_0 = \widehat{\mathrm{V}}) = \zeta \cdot \xi \cdot \gamma^n \cdot \chi. \quad (S1.11)$$

Analytical solution for the extinction probability

The probability that n infectious viruses within a target cell environment go extinct is given by

$$P_{\mathrm{E}}(Y_0 = n \cdot \widehat{\mathrm{V}}) = (P_{\mathrm{E}}(Y_0 = \widehat{\mathrm{V}}))^n, \quad (S1.12)$$

under the assumption of *statistical independence* (e.g. assuming that the competition for target cells is negligible during the onset of infection). The extinction probability for parent- and progeny virus remain identical, since the reaction rates do not change when the inhibitor efficacy is constant, as outlined in the main article.

The term P_{E} can be written as

$$\begin{aligned} P_{\mathrm{E}}(Y_0 = \widehat{\mathrm{V}}) &= \sum_{n=0}^{\infty} \mathbb{P}(Y_r = n \cdot \widehat{\mathrm{V}}|Y_0 = \widehat{\mathrm{V}}) \cdot P_{\mathrm{E}}(Y_r = n \cdot \widehat{\mathrm{V}}) \\ &= \sum_{n=0}^{\infty} \mathbb{P}(Y_r = n \cdot \widehat{\mathrm{V}}|Y_0 = \widehat{\mathrm{V}}) \cdot P_{\mathrm{E}}(Y_r = \widehat{\mathrm{V}})^n \end{aligned} \quad (S1.13)$$

In words, the extinction probability is given by the probability that n viruses are produced in a replication cycle $\mathbb{P}(Y_r = n \cdot \widehat{\mathrm{V}}|Y_0 = \widehat{\mathrm{V}})$, and that all of these viruses eventually go extinct $P_{\mathrm{E}}(Y_r = \widehat{\mathrm{V}})^n$. In the equation above, we used the *statistical independence* assumption, eqn. (S1.12), in the second equality. Since we study the eventual extinction ($t \to \infty$) and the entries of the transition matrix in our discrete-time process are constant, the equality $P_{\mathrm{E}}(Y_0 = \widehat{\mathrm{V}}) = P_{\mathrm{E}}(Y_r = \widehat{\mathrm{V}})$ holds. For brevity, we will use $\theta = P_{\mathrm{E}}(Y_0 = \widehat{\mathrm{V}})$ henceforth and solve the equation above for θ:

$$\begin{aligned}
\theta &= \sum_{n=0}^{\infty} \mathbb{P}(Y_r = n \cdot \widehat{\mathrm{V}} | Y_0 = \widehat{\mathrm{V}}) \cdot \theta^n \\
&= \mathbb{P}(Y_r = \mathbf{0} \cdot \widehat{\mathrm{V}} | Y_0 = \widehat{\mathrm{V}}) \cdot \theta^0 + \sum_{n=1}^{\infty} \mathbb{P}(Y_r = n \cdot \widehat{\mathrm{V}} | Y_0 = \widehat{\mathrm{V}}) \cdot \theta^n \\
&= (\varrho + \zeta \cdot \vartheta + \zeta \cdot \xi \cdot \chi) + \sum_{n=1}^{\infty} \zeta \cdot \xi \cdot \gamma^n \cdot \chi \cdot \theta^n \quad \text{(using eq. (S1.9) and eq. (S1.11))} \\
&= \varrho + \zeta \cdot \vartheta + \zeta \cdot \xi \cdot \chi + \zeta \cdot \xi \cdot \chi \cdot \left(\sum_{n=1}^{\infty} \gamma^n \cdot \theta^n \right) \\
&= \varrho + \zeta \cdot \vartheta + \zeta \cdot \xi \cdot \chi \cdot \left(\sum_{n=0}^{\infty} \gamma^n \cdot \theta^n \right) \\
&= \varrho + \zeta \cdot \vartheta + \zeta \cdot \xi \cdot \chi \cdot \left(\frac{1}{1 - \gamma \cdot \theta} \right) \qquad \text{(S1.14)}
\end{aligned}$$

where we used the solution of the geometric series, noticing that $\gamma \cdot \theta < 1$ as both terms γ and θ are probabilities. This shows that the extinction probability is the solution of the following quadratic problem:

$$\gamma \cdot \theta^2 - (1 + (\varrho + \zeta \cdot \vartheta) \cdot \gamma) \cdot \theta + (\varrho + \zeta \cdot \vartheta + \zeta \cdot \xi \cdot \chi) = 0 \qquad \text{(S1.15)}$$

$$\Rightarrow \theta^2 - \left(\frac{1 + (\varrho + \zeta \cdot \vartheta) \cdot \gamma}{\gamma} \right) \cdot \theta + \left(\frac{1 + (\varrho + \zeta \cdot \vartheta) \cdot \gamma}{\gamma} - 1 \right) = 0 \qquad \text{(S1.16)}$$

where we used $\varrho + \zeta = 1$, $\vartheta + \xi = 1$ and $\gamma + \chi = 1$ in the summand on the right side of the last equation. The possible solutions for θ are:

$$\theta_{1/2} = \frac{1}{2} \cdot \left(\left(\frac{1 + (\varrho + \zeta \cdot \vartheta) \cdot \gamma}{\gamma} \right) \pm \left(\frac{1 + (\varrho + \zeta \cdot \vartheta) \cdot \gamma}{\gamma} - 2 \right) \right). \qquad \text{(S1.17)}$$

The first solution $\theta_1 = 1$ is trivial, i.e. extinction is certain $P_{\mathrm{E}}(Y_0 = \widehat{\mathrm{V}}) = 1$, whereas the second solution

$$\theta_2 = \left(\frac{1 + (\varrho + \zeta \cdot \vartheta) \cdot \gamma}{\gamma} \right) - 1$$

provides some additional insights into the extinction probability. Rearranging the terms gives:

$$\begin{aligned}
\theta_2 &= \tfrac{1}{\gamma} - 1 + (\varrho + \zeta \cdot \vartheta) \\
&= \tfrac{1-\gamma}{\gamma} + (\varrho + \zeta \cdot \vartheta) \\
&= \varrho + \zeta \cdot \vartheta + \tfrac{\chi}{\gamma} \qquad \text{(S1.18)}
\end{aligned}$$

where we used $\gamma + \chi = 1$. The first part $(\varrho + \zeta \cdot \vartheta)$ is the probability that the viral replication cycle does not reach the late infected stage T_2 and the second part represents the odds of T_2 dying instead of producing virus progeny (the odds of reaction (6) firing rather than (9); the inverse of the average number of viruses being produced once stage T_2 has been reached). We have

$$\chi / \gamma = a_3 / a_6(D) = \frac{\delta_{\mathrm{T}_2}}{\mathrm{N_T} \cdot (1 - \eta_{\mathrm{PI}})}$$

as introduced in eq. (S1.5). It is evident from here, that both solutions for the extinction probability are valid and that

$$P_{\mathrm{E}}(Y_0 = \widehat{\mathrm{V}}) = \theta = \min \left(1, (\varrho + \zeta \cdot \vartheta) + \frac{\chi}{\gamma} \right). \qquad \text{(S1.19)}$$

Relation to the reproductive number

The reproductive number R_0 denotes the average number of viruses produced from a single founder virus [2].

$$R_0(D) = \sum_{n=1}^{\infty} \mathbb{P}(Y_r = n \cdot \widehat{\mathrm{V}} | Y_0 = \widehat{\mathrm{V}}) \cdot n \tag{S1.20}$$

$$= \zeta \cdot \xi \cdot \chi \cdot \sum_{n=1}^{\infty} \gamma^n \cdot n \tag{S1.21}$$

$$= \zeta \cdot \xi \cdot \chi \cdot \frac{\gamma}{(1-\gamma)^2} \tag{S1.22}$$

$$= \frac{\zeta \cdot \xi \cdot \gamma}{\chi} \tag{S1.23}$$

$$= \frac{a_4(D)}{a_1(D) + a_4(D)} \cdot \frac{a_5(D)}{a_2 + a_5(D)} \cdot \frac{a_6(D)}{a_3} \tag{S1.24}$$

where we used $(1-\gamma) = \chi$ in the second last equation. Note that the number of viruses being produced from a single founder virus is likely bimodal, i.e. in the majority of cases a single founder virus will not manage to produce any progeny, however those viruses that produce progeny will produce vast amounts of viral offspring.

We can write the infection probability for a single virus inoculum as a function of $R_0(D)$, i.e.

$$P_{\mathrm{I}}(Y_0 = \widehat{\mathrm{V}}) = 1 - P_{\mathrm{E}}(Y_0 = \widehat{\mathrm{V}}) = \max\left(0, \zeta \cdot \xi \left(1 - \frac{1}{R_0(D)}\right)\right) \tag{S1.25}$$

$$= \max\left(0, \frac{\chi}{\gamma}\big(R_0(D) - 1\big)\right) \tag{S1.26}$$

$$= \max\left(0, \frac{a_3}{a_6(D)}\big(R_0(D) - 1\big)\right), \tag{S1.27}$$

where we used the definition of $R_0(D)$ from eq. (S1.24) in the second last equality. The product $\zeta \cdot \xi$ denotes the bottlenecking process of reaching a productive compartment (productively infected cells).

Other compartments

Up until now, we have derived an analytical solution for the extinction probability conditioned that a single virus is transmitted. Similarly, we can derive the extinction probability given a single early- or late infected cell T_1 and T_2 respectively. Let the term $P_{\mathrm{E}}(Y_0 = \widehat{\mathrm{T}}_1)$ and $P_{\mathrm{E}}(Y_0 = \widehat{\mathrm{T}}_2)$ be the respective extinction probabilities for a single T_1 or T_2 cell. These probabilities relate to $P_{\mathrm{E}}(Y_0 = \widehat{\mathrm{V}}) < 1$ as follows:

$$P_{\mathrm{E}}(Y_0 = \widehat{\mathrm{T}}_1) = \min\left(1, \vartheta + \xi \cdot \chi \cdot \left(\frac{1}{1 - \gamma \cdot P_{\mathrm{E}}(Y_0 = \widehat{\mathrm{V}})}\right)\right) \tag{S1.28}$$

$$= \min\left(1, 1 - \xi \cdot \left(1 - \frac{1}{R_0(D)}\right)\right) \tag{S1.29}$$

$$= \min\left(1, 1 - \frac{a_5(D)}{a_2 + a_5(D)} \cdot \left(1 - \frac{1}{R_0(D)}\right)\right) \tag{S1.30}$$

$$\Leftrightarrow P_{\mathrm{I}}(Y_0 = \widehat{\mathrm{T}}_1) = \max\left(0, \frac{a_5(D)}{a_2 + a_5(D)} \cdot \left(1 - \frac{1}{R_0(D)}\right)\right) \tag{S1.31}$$

and

$$P_{\mathrm{E}}(Y_0 = \widehat{\mathrm{T}}_2) = \min\left(1, \chi \cdot \left(\frac{1}{1 - \gamma \cdot P_{\mathrm{E}}(Y_0 = \widehat{\mathrm{V}})}\right)\right) \tag{S1.32}$$

$$= \min\left(1, \frac{1}{R_0(D)}\right), \tag{S1.33}$$

$$\Leftrightarrow P_{\mathrm{I}}(Y_0 = \widehat{\mathrm{T}}_2) = \max\left(0, 1 - \frac{1}{R_0(D)}\right) \tag{S1.34}$$

Arbitrary initial condition/inoculum

Under the assumption of statistical independence, we have $\left(P_{\mathrm{E}}(Y_0 = \mathrm{V}\cdot\widehat{\mathrm{V}})\right) = \left(P_{\mathrm{E}}(Y_0 = \widehat{\mathrm{V}})\right)^{\mathrm{V}}$; $\left(P_{\mathrm{E}}(Y_0 = \mathrm{T}_1\cdot\widehat{\mathrm{T}}_1)\right) = \left(P_{\mathrm{E}}(Y_0 = \widehat{\mathrm{T}}_1)\right)^{\mathrm{T}_1}$ and $\left(P_{\mathrm{E}}(Y_0 = \mathrm{T}_2\cdot\widehat{\mathrm{T}}_2)\right) = \left(P_{\mathrm{E}}(Y_0 = \widehat{\mathrm{T}}_2)\right)^{\mathrm{T}_2}$, where V, T_1 and T_2 denote the number of free viruses, early-stage infected cells and late-stage infected cells. Thus, the extinction probability of any given combination of free virus V, early-stage infected cell T_1 and late-stage infected cell T_2 can be computed as

$$P_{\mathrm{E}}\left(Y_0 = \begin{bmatrix}\mathrm{V}\\ \mathrm{T}_1\\ \mathrm{T}_2\end{bmatrix}\right) = \left(P_{\mathrm{E}}(Y_0 = \widehat{\mathrm{V}})\right)^{\mathrm{V}}\cdot\left(P_{\mathrm{E}}(Y_0 = \widehat{\mathrm{T}}_1)\right)^{\mathrm{T}_1}\cdot\left(P_{\mathrm{E}}(Y_0 = \widehat{\mathrm{T}}_2)\right)^{\mathrm{T}_2}, \tag{S1.35}$$

Prophylactic efficacy

The prophylactic efficacy is defined as the reduction in infection probability per contact (compare eq. (3), main article)

$$\varphi(\widehat{\mathrm{V}}) = 1 - \frac{P_{\mathrm{I}}(\widehat{\mathrm{V}}, D)}{P_{\mathrm{I}}(\widehat{\mathrm{V}}, \varnothing)}. \tag{S1.36}$$

Rearranging yields

$$\varphi(\widehat{\mathrm{V}}) = \frac{P_{\mathrm{I}}(\widehat{\mathrm{V}}, \varnothing) - P_{\mathrm{I}}(\widehat{\mathrm{V}}, D)}{P_{\mathrm{I}}(\widehat{\mathrm{V}}, \varnothing)} \tag{S1.37}$$

$$= \frac{1}{P_{\mathrm{I}}(\widehat{\mathrm{V}}, \varnothing)}\cdot\left(P_{\mathrm{I}}(\widehat{\mathrm{V}}, \varnothing) - P_{\mathrm{I}}(\widehat{\mathrm{V}}, D)\right) \tag{S1.38}$$

where the first part is given by (compare eq. (S1.25); assuming $P_{\mathrm{I}}(\widehat{\mathrm{V}}, \varnothing) > 0$)

$$\frac{1}{P_{\mathrm{I}}(\widehat{\mathrm{V}}, \varnothing)} = \frac{1}{\zeta_\varnothing\cdot\xi_\varnothing\cdot\left(1 - \frac{1}{R_0(\varnothing)}\right)} = \frac{R_0(\varnothing)}{R_0(\varnothing) - 1}\cdot\frac{1}{\zeta_\varnothing\cdot\xi_\varnothing}, \tag{S1.39}$$

where the subscript $\varnothing$ denotes the respective term/probability for the absence of drugs. In the regimen $P_{\mathrm{I}}(\widehat{\mathrm{V}}, \varnothing) \geq P_{\mathrm{I}}(\widehat{\mathrm{V}}, D) > 0$ we have $P_{\mathrm{I}}(\widehat{\mathrm{V}}, \cdot) = \frac{\chi}{\gamma}(R_0(\cdot) - 1) = \zeta_\cdot\cdot\xi_\cdot - \frac{\chi_\cdot}{\gamma_\cdot}$. Therefore, the second part is given by

$$P_{\mathrm{I}}(\widehat{\mathrm{V}}, \varnothing) - P_{\mathrm{I}}(\widehat{\mathrm{V}}, D) = \left(\zeta_\varnothing\cdot\xi_\varnothing - \frac{\chi_\varnothing}{\gamma_\varnothing}\right) - \left(\zeta_D\cdot\xi_D - \frac{\chi_D}{\gamma_D}\right) = (\zeta_\varnothing\cdot\xi_\varnothing - \zeta_D\cdot\xi_D) + \left(\frac{\chi_D}{\gamma_D} - \frac{\chi_\varnothing}{\gamma_\varnothing}\right), \tag{S1.40}$$

where the subscript D denotes the respective terms/probabilities in the presence of drugs.

CRAs.

In the case of CRAs we have $\gamma_{\mathrm{CRA}} = \gamma_\varnothing, \chi_{\mathrm{CRA}} = \chi_\varnothing$ and $\xi_{\mathrm{CRA}} = \xi_\varnothing$ and hence the second term reduces to

$$P_{\mathrm{I}}(\widehat{\mathrm{V}}, \varnothing) - P_{\mathrm{I}}(\widehat{\mathrm{V}}, \mathrm{CRA}) = \xi_\varnothing\left(\zeta_\varnothing - \zeta_{\mathrm{CRA}}\right). \tag{S1.41}$$

We have defined $\zeta_{\mathrm{CRA}} = \frac{a_4(\mathrm{CRA})}{a_4(\mathrm{CRA})+a_1(\mathrm{CRA})}$, where $a_4(\mathrm{CRA}) = a_4(\varnothing)(1-\eta_{\mathrm{CRA}})$ and $a_1(\mathrm{CRA}) = V\left(\mathrm{CL} + (1-\eta_{\mathrm{CRA}})\frac{\beta}{\rho_{rev,\varnothing}}\cdot\mathrm{T_u}\right) - (1-\eta_{\mathrm{CRA}})\cdot a_4(\varnothing)$ and hence we get

$$\zeta_\varnothing = \frac{\beta\cdot\mathrm{T_u}\cdot\rho_{rev,\varnothing}}{\mathrm{CL}\cdot\rho_{rev,\varnothing} + \beta\cdot\mathrm{T_u}} \qquad \zeta_{\mathrm{CRA}} = \frac{\beta\cdot\mathrm{T_u}(1-\eta_{\mathrm{CRA}})\cdot\rho_{rev,\varnothing}}{\mathrm{CL}\cdot\rho_{rev,\varnothing} + \beta\cdot\mathrm{T_u}(1-\eta_{\mathrm{CRA}})} \tag{S1.42}$$

Substituting this into eq. (S1.41) we get

$$\xi_\varnothing\left(\zeta_\varnothing-\zeta_{\mathrm{CRA}}\right) = \xi_\varnothing\left(\zeta_\varnothing-\frac{1-\eta_{\mathrm{CRA}}}{\frac{1}{\zeta_\varnothing}-\frac{\eta_{\mathrm{CRA}}}{\rho_{rev,\varnothing}}}\right)=\xi_\varnothing\zeta_\varnothing\left(1-\frac{1-\eta_{\mathrm{CRA}}}{1-\frac{\zeta_\varnothing\eta_{\mathrm{CRA}}}{\rho_{rev,\varnothing}}}\right) \tag{S1.43}$$

$$= \xi_\varnothing\zeta_\varnothing\left(1-\frac{\rho_{rev,\varnothing}(1-\eta_{\mathrm{CRA}})}{\rho_{rev,\varnothing}-\zeta_\varnothing\eta_{\mathrm{CRA}}}\right)=\xi_\varnothing\zeta_\varnothing\left(\frac{(\rho_{rev,\varnothing}-\zeta_\varnothing)\eta_{\mathrm{CRA}}}{\rho_{rev,\varnothing}-\zeta_\varnothing\eta_{\mathrm{CRA}}}\right) \tag{S1.44}$$

$$= \xi_\varnothing\zeta_\varnothing\left(\frac{\left(1-\frac{\zeta_\varnothing}{\rho_{rev,\varnothing}}\right)\eta_{\mathrm{CRA}}}{1-\eta_{\mathrm{CRA}}\frac{\zeta_\varnothing}{\rho_{rev,\varnothing}}}\right)=\xi_\varnothing\zeta_\varnothing\left(\frac{(1-\upsilon)\cdot\eta_{\mathrm{CRA}}}{1-\eta_{\mathrm{CRA}}\cdot\upsilon}\right)=\xi_\varnothing\zeta_\varnothing\left(\frac{\eta_{\mathrm{CRA}}-\upsilon\cdot\eta_{\mathrm{CRA}}}{1-\eta_{\mathrm{CRA}}\cdot\upsilon}\right) \tag{S1.45}$$

$$= \zeta_\varnothing\xi_\varnothing\cdot\left(\frac{\frac{D^m}{D^m+\mathrm{IC}_{50}^m}\cdot\upsilon}{\frac{\mathrm{IC}_{50}^m}{D^m+\mathrm{IC}_{50}^m}+\upsilon\cdot\frac{D^m}{D^m+\mathrm{IC}_{50}^m}}\right)=\zeta_\varnothing\xi_\varnothing\cdot\left(\frac{D^m\cdot\upsilon}{\mathrm{IC}_{50}^m+\upsilon\cdot D^m}\right) \tag{S1.46}$$

$$= \zeta_\varnothing\xi_\varnothing\cdot\left(\frac{D^m}{\mathrm{IC}_{50}^m/\upsilon+D^m}\right) \tag{S1.47}$$

where $\upsilon=\frac{\mathrm{CL}\cdot\rho_{\mathrm{rev},\varnothing}}{\mathrm{CL}\cdot\rho_{\mathrm{rev},\varnothing}+\beta\cdot\mathrm{T_u}}<1$ denotes the probability, in the absence of drugs, that the virus is eliminated before entering a host cell. Putting the last equation and eqs. (S1.39) into eq. (S1.37) we obtain

$$\varphi(\widehat{\mathrm{V}},\mathrm{CRA})=\frac{R_0(\varnothing)}{R_0(\varnothing)-1}\cdot\frac{D^m}{\mathrm{IC}_{50}^m/\upsilon+D^m}. \tag{S1.48}$$

RTIs.

Likewise in the case of RTIs we have $\gamma_{\mathrm{RTI}}=\gamma_\varnothing$, $\chi_{\mathrm{RTI}}=\chi_\varnothing$ and $\xi_{\mathrm{RTI}}=\xi_\varnothing$ and hence the second term reduces to

$$P_{\mathrm{I}}(\widehat{\mathrm{V}},\varnothing)-P_{\mathrm{I}}(\widehat{\mathrm{V}},\mathrm{RTI})=\xi_\varnothing\left(\zeta_\varnothing-\zeta_{\mathrm{RTI}}\right). \tag{S1.49}$$

We have defined $\zeta_{\mathrm{RTI}}=\frac{a_4(\mathrm{RTI})}{a_4(\mathrm{RTI})+a_1(\mathrm{RTI})}$, where $a_4(\mathrm{RTI})=a_4(\varnothing)(1-\eta_{\mathrm{RTI}})$ and $a_1(\mathrm{RTI})=V\left(\mathrm{CL}+\frac{\beta}{\rho_{rev,\varnothing}}\cdot\mathrm{T_u}\right)-(1-\eta_{\mathrm{RTI}})\cdot a_4(\varnothing)$ and hence

$$\zeta_{\mathrm{RTI}}=\zeta_\varnothing(1-\eta_{\mathrm{RTI}}). \tag{S1.50}$$

and consequently

$$P_{\mathrm{I}}(\widehat{\mathrm{V}},\varnothing)-P_{\mathrm{I}}(\widehat{\mathrm{V}},\mathrm{RTI})=\xi_\varnothing\left(\zeta_\varnothing-\zeta_\varnothing(1-\eta_{\mathrm{RTI}})\right)=\xi_\varnothing\zeta_\varnothing\cdot\eta_{\mathrm{RTI}}. \tag{S1.51}$$

Putting together eqs. (S1.39) and (S1.51) into eq. (S1.37) we obtain

$$\varphi(\widehat{\mathrm{V}},\mathrm{RTI})=\frac{R_0(\varnothing)}{R_0(\varnothing)-1}\cdot\eta_{\mathrm{RTI}}=\frac{R_0(\varnothing)}{R_0(\varnothing)-1}\cdot\frac{D^m}{\mathrm{IC}_{50}^m+D^m}. \tag{S1.52}$$

InI.

For InIs, we have $\zeta_{\mathrm{InI}} = \zeta_\varnothing$, $\gamma_{\mathrm{InI}} = \gamma_\varnothing$ and $\chi_{\mathrm{InI}} = \chi_\varnothing$. Hence, eq. (S1.40) becomes

$$P_{\mathrm{I}}(\widehat{\mathrm{V}}, \varnothing) - P_{\mathrm{I}}(\widehat{\mathrm{V}}, \mathrm{InI}) = \zeta_\varnothing\,(\xi_\varnothing - \xi_D) = \zeta_\varnothing\left(\xi_\varnothing - \frac{k\cdot(1-\eta_{\mathrm{InI}})}{\delta_{\mathrm{PIC,T}} + \delta_{\mathrm{T}_1} + k\cdot(1-\eta_{\mathrm{InI}})}\right) \tag{S1.53}$$

$$= \zeta_\varnothing\left(\xi_\varnothing - \frac{(1-\eta_{\mathrm{InI}})}{\frac{1}{\xi_\varnothing} - \eta_{\mathrm{InI}}}\right) = \zeta_\varnothing\xi_\varnothing\cdot\left(1 - \frac{(1-\eta_{\mathrm{InI}})}{1-\xi_\varnothing\cdot\eta_{\mathrm{InI}}}\right) \tag{S1.54}$$

$$= \zeta_\varnothing\xi_\varnothing\cdot\left(\frac{\eta_{\mathrm{InI}} - \xi_\varnothing\cdot\eta_{\mathrm{InI}}}{1-\xi_\varnothing\cdot\eta_{\mathrm{InI}}}\right) = \zeta_\varnothing\xi_\varnothing\cdot\left(\frac{\eta_{\mathrm{InI}} - (1-\vartheta)\cdot\eta_{\mathrm{InI}}}{1-(1-\vartheta)\cdot\eta_{\mathrm{InI}}}\right) \tag{S1.55}$$

$$= \zeta_\varnothing\xi_\varnothing\cdot\left(\frac{\eta_{\mathrm{InI}}\cdot\vartheta_\varnothing}{1-\eta_{\mathrm{InI}}+\vartheta_\varnothing\cdot\eta_{\mathrm{InI}}}\right) = \zeta_\varnothing\xi_\varnothing\cdot\left(\frac{\frac{D^m}{D^m+\mathrm{IC}_{50}^m}\cdot\vartheta_\varnothing}{\frac{\mathrm{IC}_{50}^m}{D^m+\mathrm{IC}_{50}^m} + \vartheta_\varnothing\cdot\frac{D^m}{D^m+\mathrm{IC}_{50}^m}}\right) \tag{S1.56}$$

$$= \zeta_\varnothing\xi_\varnothing\cdot\left(\frac{D^m\cdot\vartheta_\varnothing}{\mathrm{IC}_{50}^m + \vartheta_\varnothing\cdot D^m}\right) = \zeta_\varnothing\xi_\varnothing\cdot\frac{D^m}{\frac{1}{\vartheta_\varnothing}\cdot\mathrm{IC}_{50}^m + D^m}, \tag{S1.57}$$

where $\vartheta_\varnothing = \frac{\delta_{\mathrm{PIC,T}}+\delta_{\mathrm{T}_1}}{\delta_{\mathrm{PIC,T}}+\delta_{\mathrm{T}_1}+k} < 1$ denotes the probability, in the absence of drugs that essential virus compartments get cleared intracellularly after reverse transcription and before provirus integration. Putting the last equation and eqs. (S1.39) into eq. (S1.37) we obtain

$$\varphi(\widehat{\mathrm{V}}, \mathrm{InI}) = \frac{R_0(\varnothing)}{R_0(\varnothing)-1}\cdot\frac{D^m}{\frac{1}{\vartheta_\varnothing}\cdot\mathrm{IC}_{50}^m + D^m}. \tag{S1.58}$$

PIs.

In the case of PIs we have $\zeta_{\mathrm{PI}} = \zeta_{\mathrm{PI}}$ and $\xi_{\mathrm{PI}} = \xi_\varnothing$ and hence

$$P_{\mathrm{I}}(\widehat{\mathrm{V}}, \varnothing) - P_{\mathrm{I}}(\widehat{\mathrm{V}}, \mathrm{InI}) = \frac{\chi_{\mathrm{PI}}}{\gamma_{\mathrm{PI}}} - \frac{\chi_\varnothing}{\gamma_\varnothing} = \frac{\delta_{\mathrm{T}_2}}{\mathrm{N_T}\cdot(1-\eta_{\mathrm{PI}})} - \frac{\delta_{\mathrm{T}_2}}{\mathrm{N_T}} = \frac{\delta_{\mathrm{T}_2}}{\mathrm{N_T}}\left(\frac{1}{(1-\eta_{\mathrm{PI}})} - 1\right) \tag{S1.59}$$

$$= \frac{\chi_\varnothing}{\gamma_\varnothing}\left(\frac{\eta_{\mathrm{PI}}}{(1-\eta_{\mathrm{PI}})}\right) = \frac{\chi_\varnothing}{\gamma_\varnothing}\cdot\frac{D^m}{\mathrm{IC}_{50}^m} \tag{S1.60}$$

Putting the last equation and eqs. (S1.39) into eq. (S1.37) we obtain

$$\varphi(\widehat{\mathrm{V}}, \mathrm{RTI}) = \frac{R_0(\varnothing)}{R_0(\varnothing)-1}\cdot\frac{1}{R_0(\varnothing)}\left(\frac{D^m}{\mathrm{IC}_{50}^m}\right) = \frac{1}{R_0(\varnothing)-1}\left(\frac{D^m}{\mathrm{IC}_{50}^m}\right), \tag{S1.61}$$

where we used the definition of R_0 in eq. (S1.23).

References

[1] Allen, L. J. S. *An Introduction to Stochastic Processes with Applications to Biology* (Chapman & Hall/CR, 2011).

[2] Heffernan, J. M., Smith, R. J. & Wahl, L. M. Perspectives on the basic reproductive ratio. *J R Soc Interface* **2**, 281–293 (2005).
