# Supplementary material for: Mechanistic framework predicts drug-class specific utility of antiretrovirals for HIV prophylaxis
Source: PLoS Comput Biol. 2019 Jan 30;15(1):e1006740. doi: 10.1371/journal.pcbi.1006740 (PMC6370240; doi:10.1371/journal.pcbi.1006740)
Supplement: S2 Text — (PDF) [file pcbi.1006740.s002.pdf]

Supplementary Text S2.

Sensitivity Analysis w.r.t. virus dynamics parameters

Mechanisms and parameters governing initial infection are highly uncertain and, for technical- and ethical reasons, cannot be measured in humans. In the provided software (`www.systems-pharmacology.org/prep-predictor`) it is therefore possible to freely choose model parameters. In the simulations and results presented in the main manuscript, we assumed a severe bottleneck (only few viruses reach a target-cell environment, see 'exposure model' in the main text) combined with low viral clearance rates, as immune mediated clearance of HIV may not yet be in place (resulting in a large R_0). Biological arguments for these parameter choices are given at the end of this note and in the *Discussion* (main manuscript). In terms of modelling, these conditions can be considered *conservative*, as they might under-predict prophylactic efficacy, as will become evident in this text. While the main strength of our results (and modelling in general) is to rule out certain candidates, our parameters choice could falsely rule out some candidates that *might* be efficient clinically. However, and this is the main strength, it certainly provides a more rigorous scientific basis for those drug candidates that remain promising after this screen.

Sensitivity Analysis. The prophylactic efficacy is given by (compare with eq. (3), main article)

$$\varphi(Y_0) = 1 - \frac{P_\mathrm{I}(Y_0|D)}{P_\mathrm{I}(Y_0|\varnothing)} \ , \tag{S2.1}$$

where $P_\mathrm{I} = 1 - P_\mathrm{E}$ and (compare eq. (22) main article)

$$P_\mathrm{E}\left(Y_0 = \begin{bmatrix} \mathrm{V} \\ 0 \\ 0 \end{bmatrix} \right) = \left(P_\mathrm{E}(Y_0 = \widehat{\mathrm{V}}) \right)^{\mathrm{V}}, \tag{S2.2}$$

when considering cell-free (virus only) exposure, which we will do in the following. Rearranging eq. (S1.27) in Supplementary Text S1 we get

$$P_\mathrm{E}(Y_0 = \widehat{\mathrm{V}}) = \min\left(1, 1 - \frac{a_3}{a_6(D)}\Big(R_0(D) - 1\Big)\right). \tag{S2.3}$$

When putting everything together, we have

$$\varphi(V) = 1 - \frac{1 - \left(\min\left(1, 1 - \frac{a_3}{a_6(D)}\Big(R_0(D) - 1\Big)\right)\right)^{\mathrm{V}}}{1 - \left(\min\left(1, 1 - \frac{a_3}{a_6(\varnothing)}\Big(R_0(\varnothing) - 1\Big)\right)\right)^{\mathrm{V}}} \ , \tag{S2.4}$$

for the prophylactic efficacy. The equation above has three parameters that relate to the viral dynamics, namely the basic reproductive ratio R_0, the virus inoculum V and the inverse of the expected number of viruses produced by a late infected T-cell T_2, denoted by the fraction a_3/a_6. We will discuss this parameter at the very end of this Supplementary Note. In the sensitivity analysis we will focus on $R_0(\varnothing)$ and the *average* inoculum size $\bar{V}$ since there is particular uncertainty around these parameters. Particularly, $R_0(\varnothing) = \frac{a_4(\varnothing)}{a_1(\varnothing)+a_4(\varnothing)} \cdot \frac{a_5(\varnothing)}{a_2+a_5(\varnothing)} \cdot \frac{a_6(\varnothing)}{a_3}$ will be computed as a function of the target cell density $\mathrm{T_u}$, which is a matter of debate with regard to the site of exposure (it has been argued to be small initially; discussion at the end of this note). Changes in $\mathrm{T_u}$ affect reaction propensities a_4 (target cell infection) and to a lesser extent propensity a_1 (through target-cell dependent virus clearance), compare eq. (4) and (7) in the main article.

We created a 15×15 grid, with log spaced values $1.6 \cdot 10^9 < \mathrm{T_u} < 3.2 \cdot 10^{11}$ (which results in values $1.7 < R_0(\varnothing) < 112$) and *average* inoculum sizes $0.1 < \bar{V} < 1000$. Using eq. (S2.2) we then compute the average transmission probability per exposure $\bar{P}_\mathrm{trans} \approx P_\mathrm{I}(\varnothing, \bar{V})$ in the absence of drugs and only retain those parameter sets, where $\bar{P}_\mathrm{trans}$ is in a realistic range, e.g. $0.5\% < \bar{P}_\mathrm{trans} < 5\%$ [1–4] as shown in Fig. S2.1. In total 45/225 parameter sets were retained. For these parameter sets, we computed the concentration prophylaxis profiles using eq. (S2.4) as shown Fig. S2.2.

It can be seen in Figure S2.2 (dotted profile), that our parameter choice is *conservative* with respect to predicting prophylactic efficacy: For RTIs, InIs and PIs our parameter choice denotes a worst case

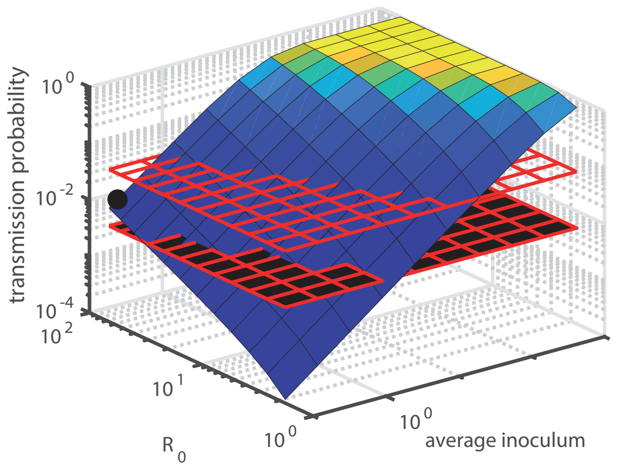

Figure S2.1: **Parameter search.** The red horizontal grids mark the lower and upper bound for the infection probability in the absence of drugs $0.5\% < P_I(\varnothing) < 5\%$. The solid dot indicates the parameters used in the main manuscript.

scenario. This means that it might under-predict prophylactic efficacy (the coloured lines are leftwards of the thick dotted line). In terms of screening for drug candidates (the practical utility of our tool), this parameter set is favourable: While some candidates may falsely be ruled out, because we under-predict their prophylactic potency, there is a much more rigorous scientific basis for those drug candidates that remain promising after this screen.

CRAs make an exception: There are parameter configurations (9/45 reasonable parameter sets) that yield EC_{50}s that exceed the ones we stated in the main manuscript. Therefore, for CRAs, we might over- or under-predict their efficacy. However, the only CRA tested, maraviroc (MRV), is not among the set of 5 compounds (EFV, NVP, ETR, RPV and DRV) which show promising concentration prophylaxis profile. For the moment, we cannot be certain about the prophylactic efficacy of MRV, but first clinical trial results indicate that it may not be potently enough on its own (see *Discussion*), which is in line with the predictions made in the main manuscript.

Biological plausibility of parameter choices. It is unclear, which physiological site is decisive for establishing irreversible infection. Some authors have argued that it is the vaginal submucosa during male → female transmission. In that context, it has been debated previously, that the target cell density at the site of viral exposure (e.g. vaginal submucosa) might initially be very low [5]. In terms of modelling, lower target cell densities strongly change (lower) reaction propensities a_4 (target cell infection) and to a lesser extent propensity a_1 (through target-cell dependent virus clearance). The net effect is that a lower proportion of viruses at the site of exposure will reach a target cell given by $\zeta = \frac{a_4}{a_1+a_4}$.

At the same time, exposure to semen and viruses, as well as inflammation may lead to the recruitment of target cells [5] shortly after exposure increasing the target cell availability. In essence, these considerations can be interpreted as follows: After breaching the mucosal barrier, there is a strong bottleneck, i.e. only a small proportion of viruses reach a target cell, denoted by ζ_{low}. Subsequent generations of viruses may encounter higher target-cell densities due to the recruitment of target cells and therefore $\zeta_{\text{low}} \to \zeta$. This additional bottleneck can be either be modelled explicitly, or implicitly by fitting an exposure distribution that captures the infection risk (as in this study). Mathematically, for the first round of replication, we would have $R_{0,\text{low}} = \zeta_{\text{low}} \cdot \frac{a_5}{a_2+a_5} \cdot \frac{a_6}{a_3} = c \cdot \zeta \cdot \frac{a_5}{a_2+a_5} \cdot \frac{a_6}{a_3}$, where $0 \le c \le 1$ denotes the bottleneck from the first replication cycle, which is caused by a low target cell density. Due to target cell recruitment, $\zeta_{\text{low}} \to \zeta$ in subsequent generations and hence $R_{0,\text{low}} \to R_0$. It is now either possible to model these processes *explicitly*, which however involves many unknowns and requires to consider a time-evolving R_0. Or, it is possible to model these aspects *implicitly* as done in the current study, by fitting an exposure model to experimentally observed infection probabilities, which may already entail the bottleneck c.

Another important aspect to consider is that it may be reasonable so assume that virus may not be cleared as efficiently shortly after the first virus exposure as compared to chronic infection. Essentially, the adaptive immune system may not yet recognise the virus during the first few days post-exposure. Current estimates of R_0 during infection are based on viral growth kinetics after the virus has become detectable

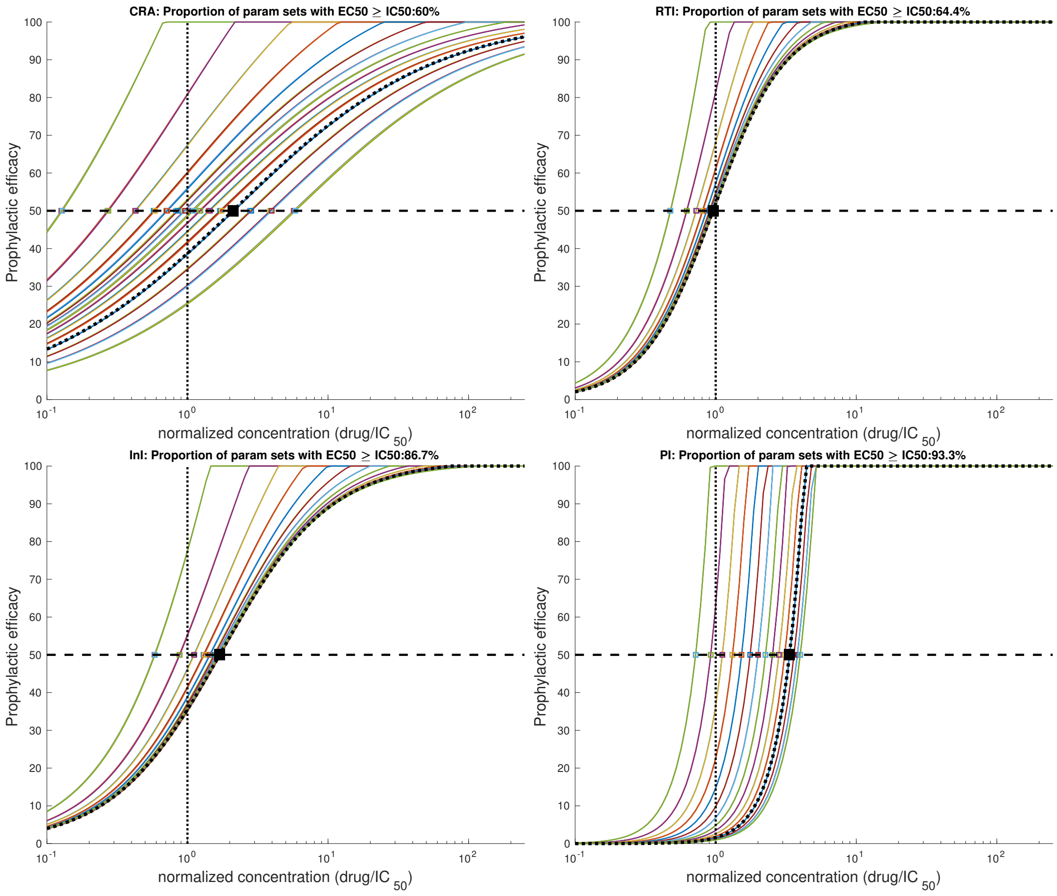

Figure S2.2: **Prophylactic efficacy estimates for different viral parameters.** The concentrations prophylaxis profiles are shown for each of the 45 parameter sets that produced reasonable infection probabilities $0.5\% < P_{\mathrm{I}}(\varnothing) < 5\%$ as shown in Fig. S2.1. The thick dotted profile indicates the profiles resulting from the parameters used in the main manuscript, whereas the coloured lines represent the profiles for the individual parameter sets. The vertical dashed line marks the IC_{50} whereas the horizontal dashed line marks the EC_{50}. We used average drug-class specific slope parameters $\bar{m}$. Upper Left: Co-receptor antagonists, $\bar{m} = 0.61$. Upper right: (non-nucleoside) reverse transcriptase inhibitors, $\bar{m} = 1.71$. Lower left: Integrase inhibitors, $\bar{m} = 1.12$. Lower right: Protease inhibitors, $\bar{m} = 2.87$.

(> 14 days after exposure). Interestingly, at this time point adaptive immune responses have developed [6] (unlike during exposure). Hence, R_0 during initial infection/immediately after exposure may be larger. In our modelling we assumed a low ability of the immune system to clear HIV, in line with the assumption that the adaptive immune system cannot yet efficiently remove HIV. This assumption results in large values for $R_0(\varnothing)$ in our study.

We did not depict the sensitivity analysis with respect to the average number of infectious viruses produced by a single late infected T-cell $\frac{a_3}{a_6(\emptyset)}$ in eq. (S2.4). Note that a_3 has previously been determined from viral decay kinetics [7] to be around 1 day^{-1}, whereas $a_6(\emptyset)$ has been determined to be 1000 per day or higher. Substituting a larger value of $a_6(\emptyset)$ into eq. (S2.4) has the following effect for all drug classes: Their *in vitro* drug potency more strongly overestimates their PrEP potency, i.e. the drugs are less efficient in preventing HIV transmission than suggested by their IC_{50}, lending further support to our statement that the *measurable in vitro* potency should not be used directly to guide clinical trial design.

References

[1] Quinn, T. C. *et al.* Viral load and heterosexual transmission of human immunodeficiency virus type 1. Rakai Project Study Group. *N Engl J Med* **342**, 921–929 (2000).

[2] Wilson, D. P., Law, M. G., Grulich, A. E., Cooper, D. A. & Kaldor, J. M. Relation between HIV viral load and infectiousness: a model-based analysis. *Lancet* **372**, 314–320 (2008).

[3] Attia, S., Egger, M., Müller, M., Zwahlen, M. & Low, N. Sexual transmission of HIV according to viral load and antiretroviral therapy: systematic review and meta-analysis. *AIDS* **23**, 1397–1404 (2009).

[4] Hughes, J. P. *et al.* Determinants of per-coital-act HIV-1 infectivity among african HIV-1-serodiscordant couples. *J Infect Dis* **205**, 358–365 (2012).

[5] Haase, A. T. Targeting early infection to prevent hiv-1 mucosal transmission. *Nature* **464**, 217–23 (2010).

[6] McMichael, A. J., Borrow, P., Tomaras, G. D., Goonetilleke, N. & Haynes, B. F. The immune response during acute hiv-1 infection: clues for vaccine development. *Nat Rev Immunol* **10**, 11–23 (2010).

[7] Perelson, A. S. *et al.* Decay characteristics of HIV-1-infected compartments during combination therapy. *Nature* **387**, 188–191 (1997).
