# Supplementary material for: Mechanistic framework predicts drug-class specific utility of antiretrovirals for HIV prophylaxis
Source: PLoS Comput Biol. 2019 Jan 30;15(1):e1006740. doi: 10.1371/journal.pcbi.1006740 (PMC6370240; doi:10.1371/journal.pcbi.1006740)
Supplement: S3 Text — (PDF) [file pcbi.1006740.s003.pdf]

Supplementary Text S3.

Relation between IC_{50} and EC_x

In this supplementary text we briefly outline the derivation of the formula that allows the computation of the prophylactic potency of drugs (e.g. EC_{50} or EC_{90}, where $x = 50, 90$ respectively) from their *in vitro* 50% inhibitory concentration IC_{50}, compare eq. (28) and eq. (32) (main article).

CRAs, RTIs and InIs.

We had previously derived

$$\varphi(\widehat{\mathrm{V}}) = \frac{R_0(\varnothing)}{R_0(\varnothing) - 1} \cdot \frac{D^m}{\mathrm{IC}_{50}^m \left(\frac{1}{\upsilon}\right) + D^m} \quad \text{(CRA)} \tag{S3.1}$$

$$\varphi(\widehat{\mathrm{V}}) = \frac{R_0(\varnothing)}{R_0(\varnothing) - 1} \cdot \frac{D^m}{\mathrm{IC}_{50}^m + D^m} \quad \text{(RTI)} \tag{S3.2}$$

$$\varphi(\widehat{\mathrm{V}}) = \frac{R_0(\varnothing)}{R_0(\varnothing) - 1} \cdot \frac{D^m}{\mathrm{IC}_{50}^m \left(\frac{1}{\vartheta}\right) + D^m} \quad \text{(InI),} \tag{S3.3}$$

which we can rewrite as

$$\varphi(\widehat{\mathrm{V}}) = C \cdot \frac{D^m}{\mathrm{IC}_{50}^m \cdot F + D^m}. \tag{S3.4}$$

Now, we define the concentration EC_x, where the prophylactic efficacy is $\varphi(\widehat{\mathrm{V}}) = x/100$, e.g. $x = 50$. Rearranging yields

$$C \cdot \frac{\mathrm{EC}_x^m}{\mathrm{IC}_{50}^m \cdot F + \mathrm{EC}_x^m} = \frac{x}{100} \tag{S3.5}$$

$$C \cdot \mathrm{EC}_x^m = \frac{x}{100} \cdot \left(\mathrm{IC}_{50}^m \cdot F + \mathrm{EC}_x^m\right) \tag{S3.6}$$

$$\mathrm{EC}_x^m \cdot \left(C - \frac{x}{100}\right) = \frac{x}{100} \cdot \mathrm{IC}_{50}^m \cdot F \tag{S3.7}$$

$$\mathrm{EC}_x^m \cdot \left(\frac{100 \cdot C - x}{100}\right) = \frac{x}{100} \cdot \mathrm{IC}_{50}^m \cdot F \tag{S3.8}$$

$$\mathrm{EC}_x^m = \frac{x}{100 \cdot C - x} \cdot \mathrm{IC}_{50}^m \cdot F \tag{S3.9}$$

and finally

$$\mathrm{EC}_x = \mathrm{IC}_{50} \left(F \cdot \frac{x}{100 \cdot C - x}\right)^{\frac{1}{m}}. \tag{S3.10}$$

PIs.

We have

$$\varphi(\widehat{\mathrm{V}}) = \frac{1}{R_0(\varnothing) - 1} \cdot \frac{D^m}{\mathrm{IC}_{50}^m} \tag{S3.11}$$

Again, defining the concentration EC_x, where the prophylactic efficacy is $\varphi(\widehat{\mathrm{V}}) = x/100$, e.g. $x = 50$, we get

$$\frac{1}{G} \cdot \frac{\mathrm{EC}_x^m}{\mathrm{IC}_{50}^m} = \frac{x}{100} \tag{S3.12}$$

$$\mathrm{EC}_x^m = \mathrm{IC}_{50}^m \cdot G \cdot \frac{x}{100} \tag{S3.13}$$

and finally

$$\mathrm{EC}_x = \quad \mathrm{IC}_{50} \cdot \left(G \cdot \frac{x}{100}\right)^{\frac{1}{m}}, \tag{S3.14}$$

as shown in the main article.
