# Supplementary material for: Mechanistic framework predicts drug-class specific utility of antiretrovirals for HIV prophylaxis
Source: PLoS Comput Biol. 2019 Jan 30;15(1):e1006740. doi: 10.1371/journal.pcbi.1006740 (PMC6370240; doi:10.1371/journal.pcbi.1006740)
Supplement: S4 Text — (PDF) [file pcbi.1006740.s004.pdf]

Drug Name	$EC_{50}(\widehat{V})$	$\overline{EC}_{50}$	Rel. diff. [%]	$EC_{90}(\widehat{V})$	$\overline{EC}_{90}$	Rel. diff. [%]
MVC	11.45	11.76	2.70	349.63	357.96	2.33
EFV	10.55	10.65	0.94	36.23	36.53	0.82
NVP	114.06	115.25	1.03	438.06	442.00	0.89
DLV	329.50	332.91	1.02	1254.58	1265.80	0.89
ETR	8.45	8.53	0.88	26.75	26.96	0.76
RPV	7.61	7.67	0.83	22.55	22.72	0.72
RAL	45.40	46.07	1.46	302.36	306.23	1.27
EVG	108.66	110.54	1.70	976.25	990.83	1.47
DTG	145.18	146.99	1.23	722.23	730.02	1.07
ATV	87.44	87.69	0.29	108.79	108.85	0.06
APV	1394.96	1400.20	0.37	1848.00	1849.32	0.07
DRV	118.32	118.57	0.22	139.24	139.30	0.04
IDV	280.80	281.29	0.17	319.71	319.81	0.03
LPV	389.69	391.18	0.38	519.09	519.46	0.07
NFV	2253.66	2263.45	0.43	3118.34	3120.91	0.08
SQV	227.29	227.78	0.21	266.66	266.77	0.04
TPV	1944.89	1950.96	0.31	2458.09	2459.55	0.06

Table S4: **Comparison of** EC_{50} **and** EC_{90}**:** $EC_{50}(\widehat{V})$ and $EC_{90}(\widehat{V})$ denote the antiviral concentrations that provide 50% and 90% prophylactic efficacy given a viral challenge with a single virus $\widehat{V}$. $\overline{EC}_{50}$ and $\overline{EC}_{90}$ denote the antiviral concentrations that provide 50% and 90% prophylactic efficacy when averaging over virus challenges sampled from the distribution for homosexual exposure (applying eq. (23), main manuscript).
