# Supplementary material for: Mechanistic framework predicts drug-class specific utility of antiretrovirals for HIV prophylaxis
Source: PLoS Comput Biol. 2019 Jan 30;15(1):e1006740. doi: 10.1371/journal.pcbi.1006740 (PMC6370240; doi:10.1371/journal.pcbi.1006740)
Supplement: S5 Text — (PDF) [file pcbi.1006740.s005.pdf]

Supplementary Text S5.

S5.1 Pharmacodynamic parameters

All considered drugs belong to the classes of co-receptor antagonists CRA, non-nucleoside reverse transcriptase inhibitors NNRTI, integrase inhibitors InI and protease inhibitors PI. Notably, for these compounds the dominant circulating agent corresponds to the bioactive form (unlike in the case of nucleoside reverse transcriptase inhibitors). We modelled the direct effects of CRA, NNRTI, InI and PI using the Emax model stated in eq. (10) (main article).

Free drug hypothesis

The Emax model requires two parameters: a hill coefficient m and an 50% inhibitory concentration IC_{50}. These two parameters have been measured *ex vivo* using single-round $CD4^{+}$ cell infection assays in 96-well plates supplemented with 50 % human serum for the majority of clinically utilized antivirals [1]. In these assays, the IC_{50} corresponds to the *total* (protein *bound* + *unbound*) concentration of the drug. It is believed under the 'free drug hypothesis' [2] that the available concentrations at the target site correspond to their *unbound* moieties [3, 4]. For CRAs the target site is extracellular, while for NNRTIs, InIs and PIs it is the intracellular space. All analyzed NNRTIs, InIs and PIs obey physico-chemical characteristics to enable the *unbound* drug to rapidly cross cellular membranes, generating an equilibrium between the *unbound* drug on either side of the cellular membrane [5]. However, since the *unbound* fraction $f_{u,\text{assay}}$ in the assay is different to the physiological *unbound* fraction $f_{u,\text{plasma}}$, the measured IC_{50} value needs to be adjusted/scaled. This adjustment is particularly relevant to some highly protein bound drugs (> 90% protein bound, see [4] for an overview).
In this Supplementary Note, we will outline the correction of the *in vitro* measured IC_{50} to its corresponding *in vivo* value.

IC_{50} value correction for protein binding

The fraction of unbound drug $f_{u,\text{plasma}}$ in the blood plasma is given by

$$f_{u,\text{plasma}} = \frac{K_d}{K_d + [PR]} \tag{S5.1}$$

where K_d denotes the dissociation constant of the drug from serum proteins and $[PR]$ denotes the concentration of serum proteins. The above equation can be reformulated to obtain

$$\frac{K_d}{[PR]} = \frac{f_{u,\text{plasma}}}{1 - f_{u,\text{plasma}}} \tag{S5.2}$$

The single-round infectivity assay is supplemented with 50 % human serum. Thus, the eq. (S5.1) for the unbound fraction of drug in the assay $f_{u,assay}$ we have

$$f_{u,assay} = \frac{K_d}{K_d + [PR]/2}. \tag{S5.3}$$

Using eqs (S5.2)–(S5.3) the relation between the unbound fraction in the assay $f_{u,assay}$ and the plasma plasma $f_{u,\text{plasma}}$ is given by

$$\begin{aligned} f_{u,assay} &= \frac{K_d/[PR]}{K_d/[PR] + 1/2} \\ &= \frac{\dfrac{f_{u,\text{plasma}}}{1 - f_{u,\text{plasma}}}}{\dfrac{f_{u,\text{plasma}}}{1 - f_{u,\text{plasma}}} + \dfrac{1}{2}} \\ &= \frac{2 \cdot f_{u,\text{plasma}}}{f_{u,\text{plasma}} + 1}. \end{aligned} \tag{S5.4}$$

The IC_{50} for the unbound drug concentrations is computed as [4]:

$$IC_{50}(\text{unbound}) = IC_{50}(\text{total}) \cdot f_u \tag{S5.5}$$

From the equation above, we can derive the following

$$\begin{aligned} IC_{50}(\text{unbound}) &= IC_{50,plasma} \cdot f_{u,plasma} = IC_{50,assay} \cdot f_{u,assay} \\ \Rightarrow &\quad IC_{50,plasma} = IC_{50,assay} \cdot \frac{f_{u,assay}}{f_{u,plasma}} \end{aligned} \tag{S5.6}$$

Using eq. (S5.4) in eq. (S5.6), we derive

$$IC_{50,plasma} = IC_{50,assay} \cdot \frac{2}{f_{u,\text{plasma}} + 1} \tag{S5.7}$$

which provides a way to translate the IC_{50} value from the single-round infectivity assays to the corresponding IC_{50} in human plasma. Table 2 in the main article summarizes pharmacodynamic- (protein adjusted IC_{50}, hill coefficient m) and pharmacokinetic parameters (fraction bound in blood plasma f_b, half life $t_{1/2}$) of all analyzed antivirals.

References

[1] Shen, L. *et al.* Dose-response curve slope sets class-specific limits on inhibitory potential of anti-HIV drugs. *Nat Med* **14**, 762–766 (2008).

[2] Smith, D. A., Di, L. & Kerns, E. H. The effect of plasma protein binding on in vivo efficacy: misconceptions in drug discovery. *Nat Rev Drug Discov* **9**, 929–939 (2010).

[3] Watkins, W. J. & Desai, M. C. HCV versus HIV drug discovery: Déjà vu all over again? *Bioorg Med Chem Lett* **23**, 2281–2287 (2013).

[4] Boffito, M. *et al.* Protein binding in antiretroviral therapies. *AIDS Res Hum Retroviruses* **19**, 825–835 (2003).

[5] von Kleist, M. & Huisinga, W. Physiologically based pharmacokinetic modelling: a sub-compartmentalized model of tissue distribution. *J Pharmacokinet Pharmacodyn* **34**, 789–806 (2007).
